# Continuing professional development (CPD) system development, implementation, evaluation and sustainability for healthcare professionals in low- and lower-middle-income countries: a rapid scoping review

**DOI:** 10.1186/s12909-023-04427-6

**Published:** 2023-07-06

**Authors:** Lisa Merry, Sonia Angela Castiglione, Geneviève Rouleau, Dimitri Létourneau, Caroline Larue, Marie-France Deschênes, Dolly Maria Gonsalves, Lubana Ahmed

**Affiliations:** 1 Faculty of Nursing, University of Montreal, Montreal, Canada; 2 SHERPA University Institute, West-Central Montreal CIUSSS, Montreal, Canada; 3 Ingram School of Nursing, McGill University, Montreal, Canada; 4 International Health Unit, School of Public Health, University of Montreal, Montreal, Canada; 5 University of Montreal Health Centre, Montreal, Canada; 6 ProNurse Project, Cowater International, Dhaka, Bangladesh

**Keywords:** Continuing professional development (CPD), Low- and lower-middle income countries, Rapid scoping review, Bangladesh, CPD system development and implementation

## Abstract

**Background:**

Policymakers and program developers in low-and lower-middle-income countries (LLMICs) are increasingly seeking evidence-based information and guidance on how to successfully develop and implement continuing professional development (CPD) systems. We conducted a rapid scoping review to map and synthesize what is known regarding the development, implementation, evaluation and sustainability of CPD systems for healthcare professionals in LLMICs.

**Methods:**

We searched MEDLINE, CINAHL and Web of Science. Reference lists were screened and a cited reference search of included articles was conducted. Supplementary information on the CPD systems identified in the articles was also identified via an online targeted grey literature search. English, French and Spanish literature published from 2011 to 2021 were considered. Data were extracted and combined and summarized according to country/region and healthcare profession via tables and narrative text.

**Results:**

We included 15 articles and 23 grey literature sources. Africa was the region most represented followed by South and Southeast Asia and the Middle East. The literature most often referred to CPD systems for nurses and midwives; CPD systems for physicians were frequently referred to as well. Findings show that leadership and buy-in from key stakeholders, including government bodies and healthcare professional organizations, and a framework are essential for the development, implementation and sustainability of a CPD system in a LLMIC. The guiding framework should incorporate a regulatory perspective, as well as a conceptual lens (that informs CPD objectives and methods), and should consider contextual factors (support for CPD, healthcare context and population health needs). In terms of important steps to undertake, these include: a needs assessment; drafting of a policy, which details the regulations (laws/norms), the CPD requirements and an approach for monitoring, including an accreditation mechanism; a financing plan; identification and production of appropriate CPD materials and activities; a communication strategy; and an evaluation process.

**Conclusion:**

Leadership, a framework and a clearly delineated plan that is responsive to the needs and context of the setting, are essential for the development, implementation and sustainability of a CPD system for healthcare professionals in a LLMIC.

**Supplementary Information:**

The online version contains supplementary material available at 10.1186/s12909-023-04427-6.

## Introduction

Around the world, continuing professional development (CPD) is increasingly expected and required of healthcare professionals in order to maintain their credentials and right to practice. CPD refers to the ongoing education and competency development by healthcare professionals beyond their initial training; the purpose is to update and advance their knowledge, skills and professional proficiency [1]. Although findings have not been consistent, a number of benefits and positive impacts have been associated with CPD, particularly in high-income countries [2]. At the individual level, in addition to acquiring new knowledge and skills (e.g., on illnesses, best practices and broader determinants that influence health and care), and improving their clinical performance (e.g., increased use of guidelines, incorporation of best practices), healthcare professionals may gain further confidence in their role, develop more positive attitudes (e.g., towards certain patient groups, their colleagues/institution) and broaden their networks; the latter may translate to reduced isolation/greater sense of belonging and increased sharing of resources and information [2]. CPD may also lead to personal growth, career advancement, including new roles and responsibilities, and scholarly achievements (e.g., publications), and hence, more commitment to the profession. At the organizational level (hospitals, educational institutions), CPD may result in new protocols and policies or curricula and pedagogical approaches for training of healthcare professionals [2]. In sum, CPD enables the healthcare workforce to evolve and better respond to patients’ needs and the ever-changing practice environment. Ultimately this may lead to better care and health outcomes [2, 3].

In many low- and lower-middle-income countries (LLMICs), CPD is not mandated and uptake and participation by healthcare professionals are limited due to access barriers [1, 4, 5]. Established CPD systems are frequently lacking in LLMICs due to funding, infrastructure and resource challenges [1]. Policymakers and program developers in these countries are increasingly seeking evidence-based information and guidance on how best to navigate these challenges and to successfully plan, develop and implement CPD systems. There are several reviews that have been conducted over the last 15 to 20 years that have examined and compared CPD systems in high-income countries, especially in Europe [6–11]. While this body of literature can offer insights and recommendations for CPD system development, implementation and sustainability, it may be of limited relevance for a LLMIC, where the social, cultural, political and economic conditions are significantly different from the contexts of many high-income (mostly Western) countries.

It is only recently that reviews, and protocols for reviews, that include or focus specifically on CPD systems in LMICs, have begun to emerge [12–20]. Magwenya et al. (2022) provide a global overview of CPD systems in different countries, including guiding frameworks and their characteristics, however, the countries examined in the review are mostly high-income countries [17]. The reviews conducted by Azad et al. (2020) and Chan et al. (2021) are restricted to CPD for nurses and pharmacists respectively; the former does not discuss CPD system development or implementation processes, while the latter is focused on only two countries (Pakistan and Jordan) [14, 19]. Guillaume et al. (2022) evaluate evidence on digital platforms used for CPD in LMICs [15] while Hill et al. (2021) aim to describe best practices and approaches, as well as facilitators and barriers when involving international collaborators from high income countries to establish and deliver CPD in LMICs [18]. Three other reviews map the continuing medical education systems (for physicians); in China, Indonesia and India [12]; in 33 countries in South-East Asia and Eastern Mediterranean regions, most of which are LMICs [13]; and in South-East Asian countries [20], respectively. Although the results across these reviews are quite extensive and informative for guiding healthcare professional CPD system development and implementation in LLMICs, the authors call for more inquiry, including additional details on the steps involved in these processes. To our knowledge, no recent review has closely looked at the development, implementation and sustainability of CPD systems for healthcare professionals (broadly defined) in LLMICs.

### Context

Bangladesh’s healthcare system faces multiple challenges in providing quality healthcare, most notably, a severe nursing shortage; it is one of the only countries where there are more doctors than nurses [21, 22]. Negative societal perceptions of the profession due to cultural, social, gender and religious factors, as well as little opportunity for career progression, have resulted in few being drawn to pursue a career in nursing [21]. Regulating training quality, especially in the private sector, has also been challenging due to the rapidity in which nursing programs have emerged across the country. Despite some attempts made towards advancing the profession, the nursing workforce remains inadequate in terms of numbers and skill level to meet population needs [21, 23, 24]. In an effort to elevate its professional status, and to increase the number of qualified and competent practicing nurses, the Bangladeshi government has committed to a series of actions to improve nurse training and development, including establishing a CPD system. This initiative is supported by the “*Empowering women through professionalization of the nursing sector in Bangladesh* (ProNurse)” project implemented by Cowater International in collaboration with the University of Montreal (Canada).

As part of the ProNurse project and to support the planning and decision-making regarding the CPD system in Bangladesh, it was requested that a review of the literature be conducted. The purpose was to identify strategies and lessons learnt from the experiences of other LLMICs that have embarked on planning, developing, implementing and/or evaluating a CPD system for healthcare professionals. This endeavor, which is reported in this paper, involved nurse researchers at the University of Montreal and the ProNurse National Nursing Specialist (DG) in Bangladesh. A technical working group comprised of key stakeholders in Bangladesh, namely, the Ministry of Health and Family Welfare (MoHFW), the Directorate General of Nursing and Midwifery (DGNM), and the Bangladesh Nursing and Midwifery Council (BNMC), were also involved. Our objective was to conduct a rapid scoping review to map and synthesize what is known regarding the development, implementation, evaluation and sustainability of healthcare professional CPD systems in LLMICs.

### Research questions

How are CPD systems for healthcare professionals developed, implemented, evaluated and sustained in LLMICs? Specifically:

- What approaches or frameworks informed CPD system development and implementation?
- Who and what processes or steps were involved in CPD system development and implementation?
- What facilitating and hindering factors influenced CPD system development, implementation and sustainability?
- What are the characteristics of CPD systems (laws/norms, monitoring methods and accreditation structures)?
- What process and outcome indicators were used to evaluate and sustain CPD systems?

## Methods

We used a scoping review methodology [25]. The Joanna Briggs Institute (JBI) guidance document for the conduct of scoping reviews [26] informed the process. Due to constraints imposed by the project timeline, rapid review methods were adopted; this consisted of restricting the number of databases and grey literature searched, applying more stringent inclusion criteria, involving only one reviewer for some of the screening steps, not contacting authors for clarifications or missing information, and limiting the stakeholder consultation to the Bangladeshi partners [27]. We did not register a review protocol. The PRISMA extension for scoping reviews was used to guide the reporting of this review (see Additional file 1).

### Database literature search strategy

The search strategy was developed in consultation with a medical librarian and with input from the research team. One team member (SC) searched three databases MEDLINE, CINAHL and Web of Science on December 13 and 17, 2021. Keywords (including truncations) and subject headings were used and combined with Boolean terms to capture four concepts: Continuing professional development (Continuing education and professional development), healthcare professionals, low to middle income countries, and program development and implementation. Searches were limited to English, French and Spanish articles published during the period of 2011 to 2021(see Additional file 2).

### Eligibility criteria

Our eligibility criteria were initially broad in order to cast a wide net. The criteria were later refined through the selection process where only the most relevant articles which aligned with the research questions would be included (see Additional file 3).

#### Population and location

We included articles where a CPD system/broad CPD program for licensed and/or regulated healthcare providers in LLMICs were described or discussed. The list of included healthcare providers was derived from the Canadian Institute for Health Information (CIHI) [28]. LLMICs were defined according to the Organisation for Economic Co-operation and Development (OECD) 2021 list of countries [29]; articles that addressed ‘low-resource settings’, even if the countries were not specified, were also considered for inclusion. Articles that focused on upper-middle income countries were excluded since the economic, social and political contexts in many of these countries are vastly different from Bangladesh.

#### CPD system

A CPD system/broad CPD program was defined as the infrastructure to support ongoing learning activities, which are provided and made available for healthcare professionals to maintain and develop a variety of knowledge and skills to meet the needs of patients and for the protection of the public [30]. We included articles that described the development, implementation and/or evaluation of a CPD system/broad CPD program, and/or described a needs assessment to inform the development or implementation of a CPD system/broad CPD program. Articles that described a CPD framework and/or its development were also eligible for inclusion. We excluded articles that described/evaluated a single or specific educational CPD activity or focused on learning activities/systems directed at students or trainees. Articles that described: the assessment/evaluation of knowledge, attitudes and practices related to a specific practice or clinical issue; the development or validation of a tool to measure CPD activities; or a global health partnership without mention of CPD system development or implementation, were also excluded. There were no restrictions regarding the research design or type of article.

### Selection of information sources

All records from the database searches were downloaded into Endnote; duplicates were removed by hand. A screening form, developed by SC with input from the team, was then used to determine eligibility. The screening process was iterative and involved multiple steps; titles and abstracts were screened first, and then full text articles. After deduplication, the first 200 titles and abstracts were screened by two reviewers (SC & LM) and discrepancies discussed. The eligibility criteria were subsequently clarified and refined based on these discussions; ongoing team meetings were held throughout the screening and selection process to ensure consistency in the application of the inclusion/exclusion criteria. The remaining titles/abstracts were screened by one reviewer (SC). Another reviewer (DL) verified 10% of excluded citations to confirm the initial screening process.

Records flagged for inclusion were independently assessed by LM, GR, and DL and a selection of these were prioritized for a full text review. To be considered for a full text review there had to be a clear indication that the article would have some information regarding the development, implementation, evaluation and/or sustainability of a CPD system/broad CPD program that could be used to inform recommendations to Bangladesh stakeholders for the development and implementation of their nursing CPD system; those with a narrow or very broad focus were assigned low priority. The short list was then reviewed by GR, DL, LM, and CL, and articles selected as priority by at least three reviewers, were retained for a full-text review. The full-text reviews were conducted by SC, who confirmed the final selection of articles for data extraction.

The reference lists were screened and a cited reference search was conducted by SC for all included articles. In order to provide updated or complementary information on the CPD systems/broad CPD programs described in the included articles, SC also conducted a targeted grey literature search using Google. Search terms included “Continuous professional development” or “CPD” and were combined with the country name to identify online information, documents and other relevant materials produced by governments associations and professional organizational bodies. Lastly, the Bangladeshi partners were consulted and they provided additional literature for consideration. Articles and grey literature sources identified through these searches and consultation were included for extraction if they met the inclusion criteria as described above.

### Data extraction process and data items

Article data were extracted by CL (*n* = 3), GR (*n* = 1), LM (*n* = 3), DL (*n* = 3), and SC (*n* = 5) and then verified by SC and LM. A data extraction form was generated using Excel, and data items were defined a priori in discussion with all reviewers. Data extracted included: country; healthcare professional(s); the CPD definition applied; the stakeholders involved in CPD development, implementation and/or evaluation; the political process; the CPD framework used; the needs assessment; the CPD system characteristics; the timeline for implementation; the implementation process; implementation barriers and facilitators identified or anticipated; evaluation process and/or outcomes; sustainability concerns/issues/strategies; the accreditation process; policies, rules, regulations and/or laws relating to CPD; recommendations for CPD system development, implementation and/or evaluation; and any other information deemed relevant to the research questions. Articles were subjectively appraised for their level of transparency and completeness, and rigor in their methods and reporting.

Data were also extracted for the grey literature. A separate Excel form with fewer items was created and one reviewer (SC) was responsible for this process. The following information was extracted: the source (website, organization); the author, document title, date and type of document; location where document was published or disseminated; web link; country; health professional(s); and information deemed pertinent to CPD system development, implementation and/or evaluation. The grey literature was subjectively appraised based on its currency, relevance, authority, accuracy and purpose [31].

### Synthesis of results

The data extracted from the articles and grey literature sources were summarized into two tables respectively. The data extracted were then combined and synthesized by country and health profession into one data matrix table in Excel. The matrix table was used to generate a narrative synthesis and several summary tables, organized according to the respective research questions (drafted by LM). Team members (CL, GR, DAL, MFD, SC) reviewed the text and tables and these were further refined based on their input. A synthesis of the findings, using PowerPoint (figures, tables, bullet points), was presented to the technical working group in June 2022. The group discussed and reviewed the results in relation to their own needs and the local context and additional revisions were made based on their feedback.

## Results

**Fig. 1:**
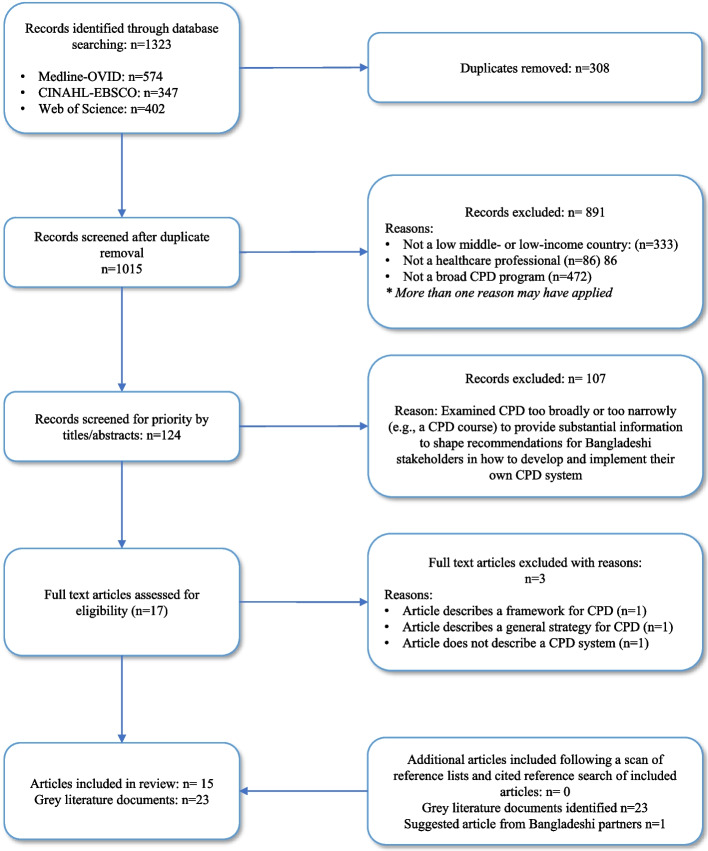
PRISMA Flow Diagram

The PRISMA flow diagram is depicted in Fig. 1. The database searches yielded 1323 records. We removed 308 duplicates and 891 records that clearly did not meet the inclusion criteria. The reviewers screened the remaining 124 records and prioritized 17 of these to be considered for full-text review. Three articles were removed during the full text review, and 14 articles were included in the review. No additional articles were included following a scan of reference lists and a cited reference search of included articles. An additional 23 grey literature sources and 1 article suggested by the Bangladeshi partners were identified and included.

### Characteristics of included articles and grey literature sources

Nine articles were published within the last five years, the other six were older publications. Eleven articles described the development and implementation of a CPD system [23, 32–39], two of these also reported on the evaluation of the CPD system [40, 41]; one article described strategies to strengthen a CPD system [42]; another article provided an overview of different approaches for implementing a CPD system in a low resource setting [5]; and two described CPD systems already in place, one of which was done with the intention of providing recommendations for improving the CPD system [43, 44]. Six regions were represented, including West Asia/Middle East (*n* = 3); South Asia (*n* = 3); West Africa (*n* = 2); South Africa (*n* = 2); East Africa (*n* = 3); and Latin America/Caribbean (*n* = 1); one paper referred to low-resource countries without specifying any location. Eight articles described CPD for nurses/midwives, while CPD for physicians and pharmacists/assistants were the focus in six and three articles respectively, and physiotherapists and dentists were each the focus in one article.

Overall, the articles were structured and clear, only one was quite difficult to read and understand [39]. The texts were sufficient to grasp the CPD system and/or its development/implementation and to have some level of confidence that the information reported was based on rigorous methods and processes. However, all of the articles lacked details on one or more aspects related to the development, implementation and/or outcomes and evaluation of the CPD system (see Additional file 4).

The grey literature varied in type, including web pages (*n* = 7), presentations (*n* = 3), guidelines/directives (*n* = 2), reports (*n* = 4), a database (*n* = 1) and other types of documents (*n* = 6). Three of the sources were published within the last five years, five were older and fifteen had no specific date of publication. The majority (*n* = 14) described one or more aspects of a CPD system (roles and responsibilities of those involved in the system, tools, guidelines, procedures, policies, and/or CPD resources and activities) [45–58]; one source had more emphasis on regulations related to CPD [59], while three others focused on the political process and stakeholder involvement [60–62], and another provided an overview of factors influencing CPD system needs, implementation, and effectiveness [63]. A couple of sources (*n* = 2) described strategies and/or recommendations for improving healthcare CPD implementation [64, 65]; one source described an international structure for CPD activities and accreditation [66], and another described an organization whose mandate is to provide support and resources (via an online platform) for CPD in LLMICs [67](see Additional file 4). Africa was the region most represented (*n* = 19), followed by South-Asia/Southeast Asia (*n* = 4) and the Middle East (*n* = 2); the countries/regions were not stated in two sources, and in one, CPD systems from several countries of different regions were discussed. Seventeen of the sources focused on or made some reference to CPD for nurses/midwives, while five referred to CPD for physicians, and two discussed CPD for pharmacists, and CPD for dentists and physiotherapists were each mentioned in one source; many of the sources referred to CPD for a mix of healthcare professionals.

Overall, the grey literature sources reported current information evidenced by the date on the document or update date found on the website, which were all within the parameter dates set for the review. All sources were deemed relevant as they provided complementary or updated information on the CPD systems reported in the articles. Accuracy of the information presented was good based on its consistency with what was reported in the articles and also across the grey literature; many had supporting references as well, and thus provided further assurance that information reported was valid. All sources were created, sponsored or authored by known and recognized organizations and institutions including national and international organizations, government ministries and professional healthcare bodies. All sources provided factual information, rather than opinion (see Additional file 4).

### CPD definition and CPD system frameworks

Across the literature, CPD incorporated different terms including most frequently, continuing education and continuing medical education. The wording of the definitions of CPD differed somewhat between the articles and sources, however there were common elements across definitions. Generally, CPD was defined as a process by which professionals maintain or acquire skills, knowledge, or competencies. CPD is viewed as a career-long responsibility and the objective is to ensure that professionals are up-to date and that their practice is safe, legal and evidence-based. It can be achieved via participation in a diversity of activities (e.g., seminars, scientific or academic work, conferences, online training modules, on-the job training) and the outcomes include personal development (self-esteem, professional advancement), strengthening of the profession/maintenance of professional standards, and improvement in the quality of care and patient/public health [23, 32–35, 37–39, 42–45, 50, 55, 56, 58, 59].

The frameworks/approaches used for guiding the development and implementation of CPD systems that were described in the literature can be broadly grouped into three categories: 1) regulatory/legislative; 2) contextual; and 3) competency/professional development. Regulatory/legislative frameworks emphasize the rules and procedures to be followed as well as the laws that govern CPD [32, 36, 44, 45, 56, 65]. Rules and procedures pertain to the healthcare professionals, the profession and to the organizations or other institutions that may be involved in delivering the CPD activities. For example, for healthcare professionals this may include the amount of CPD activities expected to be completed within a given timeframe, for the profession it could refer to the monitoring procedures, whereas for the organizations this may include the standards for accreditation. Laws refer to the CPD requirements in order to maintain licensing for a given profession.

A contextual approach stresses the importance to consider environment and system factors, including population health needs, the healthcare structure, the availability, distribution and competency level of the healthcare workforce, and the resources (financial, human, and infrastructure) and motivation at various levels (professional, system, and government) to support the implementation and sustainability of a CPD system [33, 34, 38, 41, 44, 45, 65]. Competency/professional development frameworks focus on learning and professional development principles and theories, including the competencies to be developed (standards), the level and learning needs and learning styles of the professionals, the methods that can be used to develop the competencies, and the indicators that can be used to assess for competency attainment [33, 34, 38, 45, 65]. This approach also incorporates the values, ethics and scope of practice of the profession.

The purpose of a regulatory and legislative approach is to provide structure and quality-assurance of the CPD system whereas both contextual and competency focused approaches aim to ensure the CPD system is feasible, appropriate and adapted to the local context. The three categories of frameworks/approaches are overlapping and the defining components of each are not mutually exclusively. Most articles/sources (and countries) did not overtly report using a specific framework and generally described drawing from and using various aspects from each of these approaches to guide CPD system development and implementation.

### CPD system development and implementation

Table 1 provides an overview of CPD system development and characteristics by country/region. In almost all countries, key stakeholders involved in CPD system development included the Ministry of health (or equivalent) and the healthcare professional representing bodies. Also common was the involvement of local educational institutions (universities) and hospitals and other medical services. In some countries, other governmental departments, for example the Directorate of nursing or the Ministry of labor, and/or local non-governmental organizations were also implicated; in Ethiopia and Malawi, CPD providers were involved as well. In a handful of countries, advisory and financial support were provided from international partners, for example universities in the US or from international agencies, donors and/or organizations, including professional bodies, such as the International Council of Nurses (ICN). The African Health Professions Regulatory Collaborative (ARC) provided technical assistance and guidance in a number of African countries. Information technology and CPD content expertise, especially from the World Continuing Education Alliance (WCEA), were provided in several countries/regions, including Haiti, Malawi, Lesotho, Swaziland, Liberia, Bangladesh and Pakistan.

**Table 1:** Summary of CPD system development and characteristics by country/region

Country, Region	Healthcare Professionals	Stakeholders involved	Needs Assessment	Policy, Rules, Regulations and/or Laws	CPD System (Y = yes; N = no; ? = unclear; n.s. = not stated)					
					Established	Mandatory	Credits/ points	Frequency	Monitoring system	Accreditation system
**Armenia, West Asia/ Middle East**	Physicians	Physicians, Nurses, Dentists, Pharmacists, Pharmacist assistants, EACCME, and the Armenian Ministry of Health	Needs assessment was not described There was a need to improve quality care and retention of healthcare workers	Law requires healthcare professionals to recertify to continue clinical practice	Y	Y	220 credits 140 credits (nurses)	5 yrs	Y	Y
**Jordan, West Asia/ Middle East**	Physicians and other healthcare workers	MOH, private sector, Royal Medical Services and university hospitals. Representatives from HCP groups were also consulted	A study assessed factors that influence CPD offerings, needs, practices, experiences and effectiveness. A structured self-report questionnaire was used	Mandatory participation for relicensure required by law implemented in April 2018	Y	Y	n.s	5 yrs	?	n.s
**Georgia, West Asia/Middle East**	Physicians	The president, Minister of Labor, Health, and Social Affairs, Tbilisi Medical Academy, the Professional Development Council, and the Ministry of Science and Education	No needs assessment described. It was stated that there was a particular need for improving maternal, pediatric, and perinatal care	The President made a law, then the Ministry of Labor, Health, and Social Affairs made CPD mandatory, then cancelled it	?	N	n.s	n.s	Y?	?
**Nepal, South Asia**	Physicians	IT designers and developers, local people, a group of physicians who advocate improving rural health, an international CPD content provider, medical administrators at hospitals and clinics, the Secretary of Health, Director of the Medical Council, Emergency Medicine Review, donors (Microsoft, Dtaplicity, Google, Salesforce, Github, Amazon), and NGOs (Timmy Global Health, Global Emergency Care, Handup Congo)	Interviews conducted with local physicians working in remote areas. Barriers to CPD informed the topic (emergency medicine) and the CPD infrastructure (considerations for remote access, asynchronous and with low/unstable internet, easy-to-use) and fees (free), and content should be updatable when internet is available	Nepal Medical Council mandates CPD. However, there is lack of regulatory requirement for CPD. This could change in the near future. In 2015, the medical council was working on "mandatory CPD and re-licensing requirements" but has yet to come into force	Y?	N	n.s	n.s	n.s	n.s
**Pakistan, South Asia**	Pharmacists	Pharmacists and participants in the study selected from regulatory authorities, academia and community pharmacy settings	An exploration of stakeholder's views, perceptions and practices regarding CPD in Pakistan was done using qualitative inductive methods (semi-structured interview)	CPD is not compulsory	Y?	N	n.s	n.s	n.s	n.s
**Pakistan, South Asia**	Nurses and midwives	Pakistani Nursing Council, the ICN, and the WCEA	Not described	CPD is not compulsory	Y?	n.s	n.s	n.s	n.s	n.s
**Bangladesh, South Asia**	Nurses	Partnership between American higher education and the AK Khan Healthcare Trust (a NGO) nursing faculty from Bangladesh, United States, and India, local hospital and nursing administrators, and the WCEA	Development team worked with local hospitals to define nursing staff and hospital and nursing administration educational needs. 2015 assessment of CPD showed limited implementation of CPD and in-service training, poorly coordinated and not mandatory	CPD is not compulsory	N	N	n.s	n.s	n.s	n.s
**Ghana, West Africa**	Nurses, midwives, and nursing assistants	Nursing assistants, nurses and midwives, and employer/nurse managers and educators	Not described	CPD requirements are in compliance with Part Three of Health professions regulatory bodies Act 2013 (Act 857), Nursing and midwifery council	Y	Y	Nursing Assistants: 10pts Staff nurses/ midwives—nursing/ midwife officers: 15pts Senior nursing/ midwife officer, health tutor, assistant lecturer or above: 20pts	1 yr	Y	Y
**Ghana, West Africa**	Pharmacists	Pharmacists and participants in the study selected from regulatory authorities, academia, and community pharmacy settings	Stakeholder's views were explored, including perceptions and practices regarding CPD. Qualitative inductive methods were used (semi-structured interview)	Attainment of CPD credits is compulsory	Y	Y	n.s	1 yr	n.s	n.s
**Liberia, West Africa**	Nurses and midwives	The Liberian Board of Nurses and Midwives, and the WCEA	Not described. Need based on the fact that maternal mortality ratio remains very high and quality maternal care is lower in rural areas than urban areas. There is also a lack of coordination of CPD for healthcare professionals and an absence of quality control over the training for practicing	The regulatory board for Nursing and of Midwifery offer CPD but it's not mandatory	Y	N	20 h	2 yrs	Y	Y
**Swaziland (Eswatini), South Africa**	Nurses and midwives	National Health Policy of Swaziland, the Swaziland Nursing Council, ARC, the Swaziland Post and Tele-communications Cooperation, and the WCEA	A structured questionnaire was administered to nurses and midwives to identify barriers of engagement, priority topics for CPD and the preferred learning methods. A group discussion of the findings and a literature review were then used to develop an original CPD framework to operate within the context of Swaziland	Each nurse registered with the Swaziland Nursing Council is expected to undertake CPD prior to renewing their license	Y	Y	10 h	1 yr	Y	Y?
**Lesotho, South Africa**	Nurses and midwives	MOHSW and its nursing directorate, the Lesotho Nursing Council, ARC, the Lesotho Nursing Association, NHTC, CHAL, local NGOs, and the WCEA	The MOHSW conducted a health sector human resources needs assessment in 2004	MOHSW requires professional regulatory bodies to ensure compliance in the implementation of the continuing education requirements by their members	In process	Y?	Min 12 pts	1 yr	Y	Y
**Rwanda, East Africa**	Physiotherapists	USA universities, an American NGO, Rwandan clinicians from rural & urban regions, physio-therapist faculty, and leaders of the Association of Rwandan physiotherapists	Input was gathered from stakeholders (focus groups) and a steering committee comprised of physio leaders finalized topics for the CPD courses	Law requiring CPD for licensure enacted in 2013	Y	Y	60 CPD points	2 yrs	Y	Y?
**Malawi, East Africa**	Nurses and midwives	A registrar from the NMCM, the Chief Nursing Officer from the MOH, the President of the NONM, a representative from a nurse training institution, CPD providers, ARC, a partnership between the CDC, Emory University, the Commonwealth Secretariat, and the East, Central and Southern Africa Health Community and the WCEA	An initial assessment was not described. After one year of the initial CPD program phase, monitoring and evaluation visits by the NMCM to health facilities revealed that nurses, midwives, and CPD facilitators, did not fully understand the concept of CPD, and nurse managers were not supporting staff to fulfill CPD requirements. Additional challenges included inaccessibility of CPD resources in rural posts, and knowledge deficits on the CPD documentation process The review also revealed: a need for role clarification for various stakeholders in the implementation of CPD; that there were negative attitudes toward CPD by some nursing and midwifery practitioners and that CPD activities were not "mainstreamed" by institutions and there was a need to incorporate new trends	The Nurses and Midwives Act of 1995 requires that all nurses and midwives show evidence of attending in-service education prior to licence renewal The NMCM is mandated by Nurses and Midwives Act No 16 (1995) to regulate nursing and midwifery training, education and practice CPD requirements are dependent on the type of professional and exemptions are stipulated	Y	Y	35 points	1 yr	Y	Y In process
**Ethiopia, East Africa**	All health workers	The Food, Medicine and Healthcare Administration and Control Regulation, the Federal Democratic Republic of Ethiopia MOH, CPD providers and accreditors, Healthcare Professionals, Employers, Developmental Partners and a CPD Committee comprised of: the MOH, the Regional Health Bureau, accredited professional associations, training institutions and partner members	Not described	Information on laws and norms are not stated	n.s	N	30 credits	1 yr	Y	Y
**Haiti, Caribbean**	Nurses	Nurses, Haitian MOH, Zanmi Lasante (ZL)-Partners in Health (PIH) (a NGO of providers in Haiti), HUM, head nurses, local nursing schools and local professors, and the WCEA	A needs assessment was not described. In response to the provision of new advanced care services (i.e., Intensive Care Unit) at the HUM, there was a need for nurses to quickly acquire specialized knowledge and skills to meet the complex needs of high-acuity patients in critical care units. Development of course content will also be based on the literature and the perspectives of local stakeholders	Laws and norms not discussed, but Ministry is involved in overseeing CPD	N	n.s	n.s	n.s	n.s	n.s

Abbreviations: *ARC* African health professions regional collaborative for nurses and midwives, *CDC* Centers for disease control, *CHAL* Christian health association of Lesotho, *CPD* Continuing professional development, *EACCME* European accreditation council for continuing medical education, *HCP* Healthcare professionals, *HUM* Hôpital Universitaire de Mirebalais, *ICN* International council of nurses, *IT* Information technology, *MOH* Ministry of health, *MOHSH* Lesotho ministry of health and social welfare, *NGO* Non-governmental organization, *NHTC* National health training college, *NMCM* Nurses and midwives council of Malawi, *NONM* National organization of nurses in Malawi, *WCEA* The world continuing education alliance

None of the articles or sources provided detailed accounts of the exact steps taken for developing (or updating) and implementing the CPD system and often the timeline and the order of steps were not clear. In some instances, it was only one aspect or one step of the CPD system development and implementation process that was described. Generally, it took anywhere from a few years to almost a decade to develop and implement the CPD system; some countries took longer since they initiated the process then stopped and only reinitiated again at a later time [32, 35, 37–39, 41, 42, 62]. For some countries (Haiti, Lesotho, Bangladesh), they were still in the process of planning/developing and/or implementing their CPD systems. It seems that fragmented or longer timelines may in part be due to a lack of leadership and/or motivation to implement a CPD system.

For the most part, it appears that the CPD system development and implementation process was driven by the government’s interest to improve the quality of healthcare. Certain countries, including Armenia (for physicians), Jordan (for all healthcare professionals), Ghana (for nurses and midwives), Rwanda (for physiotherapists) and Malawi (for nurses and midwives) adopted laws to enforce and regulate CPD while other countries, including Swaziland (for nurses and midwives), Lesotho (for nurses and midwives) and Nepal (for physicians) established professional norms and standards to promote CPD compliance. In Georgia (for physicians), a law was implemented but was then rescinded.

Impetus for developing and implementing a CPD system also seems to have come from the healthcare professionals, either due to the organic evolution of the profession over time, or because there was determination to actively advance the profession. The latter was most evident in African countries (Lesotho, Malawi and Swaziland), where local nursing teams led initiatives, including seeking funding and collaboration from ARC to establish their CPD systems.

Needs assessments (see Table 1) were a common initial step for assessing healthcare professionals’ attitudes towards CPD, their current involvement in CPD activities, their interest and willingness to participate (or continue to participate), and preferences/needs for CPD (priority topics and format), as well as barriers to participation. Assessment of key stakeholders’ (healthcare institutions, healthcare professional associations, universities) perceptions and priorities for CPD, was also often part of this process. Input from healthcare professionals and stakeholders was gathered either from a self-report questionnaire or directly via interviews or focus groups. Reviewing existing policy documents (locally and internationally) and consulting the scientific literature was also sometimes done in conjunction with a needs assessment.

Other steps in CPD system development and implementation noted across the literature [5, 37, 38, 42, 45, 52, 62, 64] were: developing a framework; outlining the specific CPD requirements (e.g., number of hours or credits to complete, timeframe for completing CPD activities, activities considered eligible) and drafting a policy; planning the financing (applying for grants); specifying the roles and responsibilities of each stakeholder in the CPD system; determining who and how CPD will be monitored and mobilizing key stakeholders accordingly; defining the outcomes and process for evaluating the CPD system; creating a system and the materials (guidelines, forms, etc.) for recording and logging CPD (which may or may not be directly linked to licensing renewal and may be done through an online or paper-based system); identifying and/or designing and offering CPD activities and materials (online or in-person) and if needed, providing training to CPD providers; developing and implementing training for healthcare professionals (e.g., how to use an application) and for those monitoring CPD activities (e.g., verification procedures); mounting and launching a communication strategy (websites, brochures, radio, site visits, texts) to raise awareness and foster involvement and compliance in the CPD system for all those concerned; and planning and putting in place an accreditation structure for CPD providers and activities, including eligibility criteria and the procedures for obtaining accreditation.

The process of CPD development and implementation was iterative and gradual in many cases. For example, Nepalese physicians were asked to provide feedback pre- and post-development of a CPD mobile application through three design cycles. Similarly, in Liberia the CPD platform and mobile application for nurses and midwives were pilot tested before being fully launched. In Lesotho, the CPD system for nurses and midwives was pilot tested and received feedback both from regional peers and technical experts at two ARC learning sessions held during the project period. The implementation was also done in a staged process across districts, and CPD was initially voluntary and then became mandatory over time. In Rwanda, CPD courses for physiotherapists were initially delivered by US academics and clinical experts and Rwandan co-instructors, and then the latter progressively took over. In Haiti, the intention was to implement a specific CPD program (a series of modules) for nurses that could then be scaled-up and/or used as a model for the development of other programs.

### Barriers, facilitators and CPD system sustainability

A number of barriers and facilitators to CPD development and implementation on the delivery and user-ends, at both the individual and system levels, were observed across countries (see Table 2). At the system level, key barriers include a lack of funding, structures and qualified human resources to coordinate and manage the CPD system, which are exacerbated when there is no support from the government or healthcare professional bodies [32, 37, 38, 41, 42, 63]. At the individual level, participation and adherence to CPD are affected by limited technical skills, competing demands (e.g., family obligations), and when there is a disinterest in, or negative views of CPD, among healthcare providers [37, 40, 42, 44, 63]. Workplace (e.g., scheduling, low salaries) and environmental factors (e.g., limited electricity, difficult transport) further contribute to adherence issues [32, 35, 37, 38, 40, 41, 44, 63]. To address these challenges, leadership, financial investment, and the mobilization of material and human resources, including the training of personnel, all at the local level, are required [37, 38, 42, 62]. Making CPD mandatory, offering a diversity of options for CPD, that are easily accessible and available at low-cost, and ensuring work environments are supportive, are suggested strategies to promote CPD uptake [32, 33, 35, 40, 41, 44, 62, 63]. Active promotion and marketing, and providing incentives, may also alter healthcare providers’ attitudes and further lead to participation in CPD activities [37, 44, 63]. Overall, sustainability of a CPD system requires buy-in and support from a diversity of stakeholders, consistent funding and resources, and the establishment of robust regulation, monitoring and accreditation structures [37, 38, 41, 42, 45, 62, 63]. The system should also be dynamic wherein modifications and improvements can be introduced over time.

**Table 2:** Barriers and facilitators to CPD system development and implementation^a^

Barriers	Facilitators
***System*** • Lack of government funding to support the system/dependence on grants or donor support • No buy-in from stakeholders • Lack of human resources, time, skills and/or capacity to develop and implement and maintain the CPD system, including CPD activities and resources • Poor coordination and structure, including technological capacity, within the system • Confusion and misunderstandings among stakeholders regarding their roles and responsibilities • Absence of quality control • Translated CPD materials not available in local language • CPD materials available are not adapted to local context ***Individual*** • Low motivation and interest in CPD and/or a lack of understanding of the concept from professionals • A lack of technical skills • Insufficient time to participate in CPD activities (workload, family obligations, especially for women) • Difficulties tracking hours/credits completed and/or completing the documentation process for CPD recognition ***Environment (hospital, community)*** • Unsupportive employers and administrators (no time allotted for CPD) • Cost of CPD activities (low salaries) • Incompatible scheduling or inadequate availability of CPD activities and resources • Difficult access to CPD activities (transport, road conditions and lodging, particularly for those in non-urban areas) • Limited internet connection or electricity and technical issues (e.g., old operating systems)	***System*** • Leadership • Key stakeholders actively support and are involved in the development and implementation process from the onset • Having ongoing marketing and sensitization including promotion of CPD benefits • CPD is compulsory • Funding • The system builds from existing structures and policies • The system is structured, efficient and properly resourced, including monitoring and accreditation processes ○ CPD monitoring linked to the licensing registry/database; ○ core courses are offered; ○ human resources are available and trained to maintain websites, forms, databases and to conduct monitoring and accreditation; ○ guidelines exist for each process; ○ CPD facilitators or coordinators are available and trained support and oversee CPD process at the ground level; ○ roles of stakeholders are clearly delineated; ○ ongoing technical support is provided • Outcomes for CPD system evaluation are determined and defined a priori • The system is harmonized within a larger CPD system (i.e., recognition of CPD activities already accredited by other associations or organizations internationally) • There is capacity to develop and offer suitable CPD activities • A diversity of CPD activities are available and recognized for credits/hours (e-learning and in-person; local and international level; from different organizations and institutions; theoretical, practical, or professional in content) • CPD activities are easily accessible, low cost/free, and offered at convenient times (e.g., online repository where content can be downloaded; transport support provided or activities offered onsite or in multiple locations; targeted, prescribed CPD activities/programs are offered) • CPD activities are straightforward to complete • CPD activities are tailored to learning needs and are relevant and up to date according to practice and population health needs ***Individual*** • Positive attitudes toward CPD ***Environment (hospital, community)*** • Employers and administration are supportive (protected time, culture and environment that values CPD) • Incentives to complete CPD are provided (promotion, salary increases)

^a^ Summary based on experiences and recommendations found across the literature; *CPD* Continuing professional development

### CPD system characteristics

#### Laws and/or norms and standards

CPD is mandatory and required for re-licensing in Armenia (physicians), Jordan (physicians), Ghana (nurses, midwives, pharmacists), Rwanda (physiotherapists), Swaziland (nurses and midwives) and Malawi (nurses and midwives), and it’s not (or yet) compulsory in Georgia (physicians), Nepal (physicians), Pakistan (nurses, midwives and pharmacists), Liberia (nurses and midwives), Haiti (nurses), Ethiopia (all healthcare professionals) and Bangladesh (nurses). In Lesotho (nurses and midwives) it was in the process of becoming mandatory (see Table 1).

#### CPD monitoring

Different systems are used for quantifying and monitoring CPD, either hours or credits/points; requirements usually differ by profession, with the number of hours or credits being highest for physicians (see Table 1). For nursing, Armenia requires 140 credits/5 years; Ghana, 10 points/year; Malawi, 35 points/year; Liberia, 20 h/2 years; Swaziland 10 h/year; and Lesotho 12 points/year. CPD formats and content available and considered eligible for CPD credit, vary. Formats include in-person or distance-learning courses, e-learning materials, seminars, online platform/mobile applications (e.g., WCEA), self-directed learning activities, tailored programs, workshops, conferences, academic events, formal education, in-service trainings, receiving coaching or mentoring, research-related activities (e.g., publishing) and other types of activities (e.g., participating in policy development). Content comprises theoretical knowledge, practical, clinical training, and/or professional development (e.g., communication, ethics). In several countries, CPD activities have to be accredited or approved by the ministry of health and/or professional representing bodies to be accepted for CPD recognition; Armenia also accepts activities accredited by the European Accreditation Council for CME (EACCME) and the American Medical Association (AMA). Some countries (e.g., Armenia, Ghana, Rwanda) stipulate that to fulfill requirements there must be certain types of CPD activities completed (e.g., practical skills, and theoretical knowledge) while in other countries there is compulsory content (e.g., HIV/AIDS in Lesotho) or modules or courses (e.g., Malawi) that must be followed. Lesotho also has some restrictions on the percentage of CPD activities that can be self-directed learning or in-service training.

The process for monitoring differs across countries as well. Armenia put in place a dedicated, centralized center for monitoring which also maintains a registry of all healthcare workers. In Swaziland, Lesotho, Ghana and Malawi, for nurses and midwives, and in Rwanda for physiotherapists, professionals are required to maintain a logbook and have their CPD activities verified by their employer or the CPD coordinator at their institution and this information is then forwarded to the professional regulatory body. In contrast, in Ethiopia, the ministry of health (a designated case team) is responsible for overseeing the monitoring. Information and documentation that must be recorded and submitted may include: the description of the CPD activity (type, content, location and provider), date of completion, the number of hours, the learning objectives and outcomes, and/or a certificate as proof of completion of an activity. The period for monitoring is most often done annually (Ethiopia, Malawi, Lesotho, Ghana), in Rwanda and Liberia it’s done every other year, and in Jordan and Armenia, it’s conducted every five years. Armenia and Lesotho mentioned disciplinary action for non-compliance (in Armenia a test must be passed if less than 70% of credits are not obtained; in Lesotho the license may be suspended, or the individual may be required to pass an exam or practice under supervision). In Malawi, Ghana and Lesotho, nurses and midwives may be exempt for CPD, for example, if a clinician is retired or not actively practicing the profession.

#### CPD accreditation

Regarding accreditation and approval of CPD activities and resources, in Armenia, it’s the centralized center that is responsible for this task (as mentioned already, they also accept activities accredited by the EACCME and AMA), whereas in other countries it’s either the professional regulatory body (e.g., Lesotho, Rwanda, Malawi, Liberia) or the Ministry of health (e.g., Georgia, Ethiopia) who oversees this process; in Ethiopia, however, the ministry delegates the responsibility to a committee comprised of government and healthcare officials. In some countries CPD providers can also be accredited (e.g., Lesotho, Liberia, Rwanda, Malawi, Ethiopia). In Rwanda the professional association is directly involved in the development of CPD courses.

Some of the criteria used to accredit/approve activities, resources and CPD providers include: relevance to current or future practice; recency of the content; scientific soundness (i.e., evidence-based); has a measurable outcome (e.g., diploma); potential for bias or conflict of interest; qualifications of those who developed and/or who are delivering the CPD activities; learning objectives; teaching and learning methods; accessibility and affordability; time needed to complete the activity; adequacy and appropriateness of the resources to deliver the training; and learning assessment procedures (to determine whether learning objectives have been met). The amount of credits or points allotted for different activities may vary. Activities within or outside of the country may be eligible for accreditation (e.g., Lesotho, Armenia). To obtain accreditation, an application usually must be submitted to the administering body and renewal is required to maintain the status; in Lesotho renewal is required after two years. In Ethiopia, quality assurance checks of CPD providers are done by the CPD committee. A summary of CPD accreditation criteria is provided in Table 3.

**Table 3:** CPD accreditation criteria and indicators for evaluation^a^

*CPD accreditation criteria*	*Indicators for CPD system evaluation*
• Relevant • Recent • Evidence-based • Accessible and affordable • Non-biased/no conflict of interest • CPD developers and/or providers are qualified • Objectives and teaching and learning methods are suitable and adapted to the learners • Has a measurable outcome • Time required to complete the activity is reasonable • Resources are adequate for delivery of the training • Learning assessments are appropriate	• Number of professionals participating in CPD activities and meeting requirements • Frequency, type and quantity of CPD activities and resources accessed • Who and where were CPD activities offered • Satisfaction with CPD activities and resources • Challenges experienced while accessing CPD activities and resources • Knowledge, skills and competency levels of professionals • Attitudes towards CPD • Monitoring process (challenges experienced, number of professionals verified) • Quality assurance (CPD providers) • Retention of healthcare professionals • Quality of healthcare • Patient/population health outcomes

^a^ Summary based on data extracted from the articles and grey literature sources

*CPD* Continuing professional development

### CPD system evaluation

The evaluation of the CPD system was not explicitly described in most resources or articles. Across countries, evaluation tended to be the responsibility of the government and/or of the professional representing body; outcomes considered, frequency of evaluations, and methods of evaluation varied. For CPD systems using the WCEA platform (e.g., Liberia, Pakistan, Malawi), an evaluation component is embedded within the system and provides data on the number of users, the frequency of use, and which courses were accessed and completed. In Liberia, in addition to the WCEA system, progress learning of nurses was also evaluated through a mobile service managed by the Ministry of health and through visits from reproductive health supervisors; quality of care was assessed every three years.

In Nepal, improvements of the CPD mobile application for physicians were informed by data generated through the integrated user-tracking and feedback system. There were also plans to assess the impact of the application on clinical practices, professional development, and views on CPD by administering a questionnaire one year post-implementation. For the CPD program developed for nurses in Bangladesh, participants’ understanding and application of knowledge and skills were assessed directly through quizzes, clinical laboratories and debriefing sessions and indirectly through feedback collected from their nursing directors; data on nurses’ perceptions of the program were also collected. A recommendation was also made to gather data on health outcomes in order to assess impacts over the long term.

In Malawi, a taskforce was created to evaluate the CPD system through site visits to healthcare facilities. They gathered data from the nurses (e.g., CPD points completed) and from those monitoring and facilitating CPD (e.g., understanding of CPD and role) and also on the quality and dissemination of the CPD materials. In Ethiopia, the case team, put in place by the Ministry of health, is responsible for overseeing the evaluation of the CPD system (all healthcare professionals), which includes ensuring that the CPD provider list is up to date, quality assurance (CPD providers and content) is adhered to, and that overall, CPD is implemented across the various professions. They also provide general support to the CPD committee who is responsible for regulation.

In Lesotho and Swaziland, evaluations are conducted every other year via a random distribution of a survey to 5–10% of nurses and midwives in each district; some of the data gathered included: level of satisfaction, attitudes toward CPD, barriers in accessing activities, type of activities completed, compliance with CPD requirements and recommendations for improvement. Surveys were also distributed to nurses who were responsible for verifying logbooks; data collected included number of logbooks verified, attitudes toward CPD, challenges and experiences with the verification process, and recommendations for improvement. In Lesotho, statistics (e.g., number of individuals attaining required points, number of logbooks submitted and accurately verified) were also generated on an annual basis. In Rwanda, the evaluation of the series of CPD courses developed for physiotherapists was done concurrently with implementation. It involved site visits, pre-and post- knowledge and feedback surveys with participants, instructors’ reports and debriefing with stakeholders. Lastly, in Haiti, evaluation of the CPD program (series of modules) for nurses is planned and the elements to be assessed include: retained knowledge, perceived autonomy, job satisfaction and intention to stay, and turnover rates within the profession. Methods will involve self-evaluations, structured observations and pre-post tests. A summary of indicators for CPD system evaluation is reported in Table 3.

## Discussion

Our objective was to conduct a rapid scoping review to map and synthesize what is known regarding the development, implementation, evaluation and sustainability of healthcare professional CPD systems in LLMICs in order to inform stakeholders’ planning and decision-making for the development and implementation of a CPD system for nurses in Bangladesh. In summary, a framework, leadership and buy-in from key stakeholders, access to resources and a clearly delineated plan that is responsive to the needs and context of the setting, are essential for the development, implementation and sustainability of a CPD system for healthcare professionals in a LLMIC.

The review does not point to any particular framework that should be adopted when developing and implementing a CPD system in a LLMIC but rather it suggests that it should incorporate a regulatory perspective, as well as a conceptual/theoretical lens, and should consider contextual factors. As remarked by others, regulation, standards and/or a legal framework, can provide CPD legitimacy, while a conceptual approach (to inform the CPD goals and guide the methods and activities), that aligns with the local/profession’s culture, attitudes and values, optimizes effectiveness of the system [1, 13, 17, 19, 68]. Magwenya et al. (2022) further specify that the use of validated theories (e.g., adult learning, behavioural change, reflective practice, problem-based learning, quality improvement) can lead to more efficacious CPD activities [17]. It has also been previously emphasized that successful CPD system implementation and sustainability can only be achieved if the local environment is taken into account, in particular population health needs and the existence of support for a CPD system [1, 3, 14, 19].

It is clear from the literature that leadership and buy-in from stakeholders, including government ministries and healthcare professional bodies and associations, are crucial for the establishment of a sustainable CPD system. As shown in the review, a number of challenges exist in LLMICs that render the CPD system development and implementation much more complex, namely limited financing, technological barriers, a lack of human resources and structures to maintain a CPD system, negative attitudes, and a dearth of CPD materials and activities that are relevant to the context and available in the local language. As highlighted by others, and also in this review, government level engagement can ensure resources, including financing, are mobilized, and can provide support for setting standards; they may also pay a role in enforcement [1, 10, 13, 17]. Vakani et al. (2022) observed, however, that government involvement and effectiveness are often related to political stability within a country, and thus the larger context must be considered as well [13]. As for healthcare professional bodies, it has been shown that they are often well-positioned to identify strategies to help navigate some of the implementation challenges, and that championing from the profession can enhance relevancy and promote positive attitudes and uptake of CPD [1, 13, 14, 18, 19]. In the context of nursing, particularly in Bangladesh, the implication of nurse leaders may also be empowering [19] and strengthen societal perceptions of the profession, which in turn may further aid in securing support and resources for a CPD system.

The review reveals that partnerships with high-income countries or international organizations, are common. Involvement of international partners can have benefits by providing expertise, knowledge, skills and funding, but a Western bias, and sustainability over the long-term are significant issues. The results emphasize that capacity-building at the local level, including the training and development of local CPD providers, is vital for ensuring sustainability; this notion is supported by others as well [12, 13]. In addition, Hill et al. (2021) underscore the importance of collaborations being genuine, founded on principles of cultural humility, trust and equity, and reflexivity, with commitment for the long-term [18]. Two international actors, the ARC initiative and the WCEA, were identified in the review as key collaborators and resources that may be accessed for CPD system development and implementation support in LLMICs. The ARC has played a meaningful role in providing technical assistance and tools to a dozen of African countries to improve and advance their nursing and midwifery CPD systems. They have created a number of resources, including a toolkit [69] which includes a step-by-step guide for developing and implementing a CPD system; a survey questionnaire that may be used for conducting a needs assessment; example of how to approach evaluation; a list of criteria for accrediting CPD providers; and a list of web links for accessing additional materials, activities and tools. They have also developed an online library of continuing education content [70]. The WCEA has also partnered with a number of countries and international stakeholders; they offer e-health and m-health solutions in order to address capacity and relevance issues related to CPD delivery [71]. More recently, an initiative funded by the Japan International Cooperation Agency (JICA), provides additional opportunities for knowledge and resource sharing between countries of the Asia Pacific Action Alliance on Human Resources for Health, through their ongoing workshops on CPD system development and implementation for nurses [72]. The workshops are forums that offer concrete solutions and tools for participating LLMICs seeking to establish or improve their CPD systems.

Regarding the plan for developing and implementing a CPD system, no paper or study was identified that provided an explicit overview of the steps to follow. However, taken together the review indicates that the process should include: establishment of leadership; a needs assessment; development of a framework; drafting of a policy (based on the framework), which details CPD requirements and a monitoring and regulation strategy, including an accreditation mechanism; a financing plan; identification and production of appropriate CPD materials and activities; a communication strategy; and an evaluation process. The process is often iterative and steps may vary depending on the context. These findings are coherent with recommendations from the WHO, the United States Agency for International Development (USAID), the ARC toolkit, as well as recent reviews, which all highlight one or more of these as key elements to CPD system development and implementation in a LLMIC [1, 3, 12–14, 17, 73]; they also iterate the importance of having a strategy, i.e., an outline of steps to be followed, towards developing and implementing a CPD system. In terms of the financing plan, what emerged as crucial, is ensuring that funding is not short term (i.e., not dependent on grants or international aid). The recent review by Magwenya et al. (2022) also recommend including a code of ethics, as well as clear guidelines concerning the involvement of the pharmaceutical industry to avoid commercial influence [17]. They also advocate for low-cost activities so that healthcare providers do not bear an unreasonable burden in order to participate in CPD, which is consistent with the findings in this review. With regards to monitoring approaches, and the content and delivery methods of CPD, there is no consensus, but rather it is proposed that these should be determined according to local needs and setting to ensure effectiveness [1, 3, 12–14, 17, 73]; making CPD mandatory, however, does appear to be an effective strategy for ensuring CPD uptake. For accreditation, clear standards need to be put in place [12, 13, 17], while for CPD system evaluation, a number of indicators can be applied; Magwenya et al. (2022), however, emphasize that healthcare provider behavioural changes and patient outcomes are key items to measure over the long term. To further inform this process, future research that provides a longitudinal view of CPD system implementation in LLMICs, including ongoing evaluation to assess for sustainability, is warranted.

Lastly, the findings of the review served as the basis for the development of an action plan for the nursing CPD system development and implementation in Bangladesh (see Additional file 5). This action plan is currently being implemented and includes a timeline and a number of detailed steps according to the needs and context in Bangladesh. The DGNM and the BNMC will play an important leadership role. This action plan adds to the repertoire of existing resources, and can be used as a template for other LLMICs planning, developing and implementing a CPD system.

### Strengths and limitations

Due to the rapid nature of the review and the prioritization process used to select articles and sources, not all relevant literature may have been included. Details were not always clear or were piecemeal across the articles and sources, and so it’s possible that there are some inaccuracies in the data extracted and reported. We also did not conduct a widespread check to verify consistency of the data with official country documents or sources. There was no evaluative component to our process, therefore conclusions regarding the best approaches for developing, implementing and evaluating a CPD system cannot be drawn. However, we included data from a range of different countries and regions, our process was rigorous, and the summary tables and the action plan provide a broad, comprehensible overview of the various considerations and steps involved when developing, implementing and evaluating a CPD system in a LLMIC, and thus can be useful resources for others undertaking such an endeavour in the future.

## Conclusion

CPD can strengthen networks, improve motivation and be empowering for healthcare professionals in LLMICs, especially for those working in more isolated and rural regions [3]. The positive effects of CPD in turn can translate to retention and higher quality care [19]. To effectively develop, implement and sustain a CPD system in a LLMIC, leadership, especially from the healthcare professionals, a framework, funding, CPD materials and activities that are accessible and responsive to local needs, and the establishment of robust regulation, monitoring and accreditation structures and an evaluation mechanism, are essential.

## Supplementary Information

**Additional file 1.** Preferred Reporting Items for Systematic reviews and Meta-Analyses extension for Scoping Reviews (PRISMA-ScR) Checklist.

**Additional file 2.** Search Strategy for Medline.

**Additional file 3. **Refinement of Inclusion and Exclusion Criteria.

**Additional file 4.** Characteristics of Included Articles and Grey literature.

**Additional file 5.** Bangladesh TWG-CPD action plan and timeline, July 2022.

## Data Availability

All data generated or analyzed during this study are included in this published article and its supplementary information files.
