# Supplementary material for: Continuing professional development (CPD) system development, implementation, evaluation and sustainability for healthcare professionals in low- and lower-middle-income countries: a rapid scoping review
Source: BMC Med Educ. 2023 Jul 6;23:498. doi: 10.1186/s12909-023-04427-6 (PMC10324177; doi:10.1186/s12909-023-04427-6)
Supplement: Supplementary file 2 — Additional file 2. Search Strategy for Medline. [file 12909_2023_4427_MOESM2_ESM.docx]

**Additional file 2: Search Strategy for Medline**

| Concepts | Concept 1 :  Continuing professional development | Concept 2 :  Healthcare professionals | Concept 3:  Low to middle income countries | Concept 4:  Program development and implementation |
| --- | --- | --- | --- | --- |

| **Concept 1 : equation with descriptors (1)** |  | education, continuing/ or education, dental, continuing/ or education, medical, continuing/ or education, nursing, continuing/ or education, pharmacy, continuing/ or inservice training/ |
| --- | --- | --- |
| **Concept 1 : equation with keywords (2) (Title, abstract, keyword heading, keyword headings word** |  | ((continuing OR Inservice OR Post-Registration) ADJ2 Education)  OR  (("On the job" OR workplace OR Inservice) ADJ2 Training)  OR  (("life long" OR lifelong OR ongoing OR activit*) ADJ2 learning) |
| **3=1 OR 2** |  | OR |
| **Concept 1 : equation with descriptors (4-6)** | 4  5  6 | Staff development/  Exp professional competence/  capacity building/ |
| **7= 4 OR 5 OR 6** |  |  |
| **Concept 1 : equation with keywords (8)** |  | (professional ADJ2 (development OR growth))  OR  ((professional OR clinical) ADJ2 (knowledge OR skill* OR competenc*))  OR  (competenc* ADJ2 development)  OR  (workforce ADJ2 (development OR competenc* OR knowledge OR skill*))  OR  (skill* ADJ2 acquisition)  OR  (job ADJ2 performance)  OR  (capacity ADJ2 build*)  OR  "Staff development" |
| **9= (7) OR (8)** |  |  |
| **10=(3) AND (9)** |  |  |
| **Concept 1 : equation with keywords (total) (11)** | OR | "continuing professional development" |
| **Concept 1 : mixed equation (12)** | **OR** | **10 OR 11** |
| **Concept 2 : equation with descriptors (13)** |  | health personnel/ or allied health personnel/ or dental assistants/ or dental hygienists/ or emergency medical technicians/ or licensed practical nurses/ or nursing assistants/ or exp anesthetists/ or audiologists/ or "coroners and medical examiners"/ or exp dental staff/ or exp dentists/ or exp infection control practitioners/ or exp medical staff/ or exp nurses/ or exp nursing staff/ or exp nutritionists/ or exp occupational therapists/ or exp optometrists/ or exp pharmacists/ or exp physical therapists/ or exp physicians/ or exp social workers/ |
| **Concept 2 : equation with keywords (14)** |  | ((Health* adj2 (professional* or personnel or Staff or worker* or provider* Employee*)) or Audiologist* or Chiropractor* or (Dental adj2 (assistant* or hygienist*)) or Nurse* or Physician* or Physiotherapist* or Dentist* or Dietician* or Doctor* or "Genetic Counsellor*" or (Medical adj2 ("laboratory technologist*" or "radiation technologist*")) or Midwi* or Nutritionist* or (Therapist* adj2 (occupational or respiratory)) or Optician* or Optometrist* or Paramedic* or Pharmacist* or Psychologist* or "Public Health Professional*" or "Speech-Language Pathologist*" or "Social Worker*").ab,kf,kw,ti. |
| **Concept 2 : mixed equation (15)** | **OR** | **(13 OR 14)** |
| **Concept 3 : equation with descriptors (16)** |  | developing countries/ or exp africa/ or exp caribbean region/ or exp central america/ or exp latin america/ or exp mexico/ or exp south america/ or exp asia, central/ or exp asia, southeastern/ or exp asia, western/ or exp china/ or exp "democratic people's republic of korea"/ or mongolia/ or albania/ or "bosnia and herzegovina"/ or kosovo/ or "republic of north macedonia"/ or moldova/ or montenegro/ or "republic of belarus"/ or ukraine/ or exp transcaucasia/ or comoros/ or madagascar/ or mauritius/ or sri lanka/ or indonesia/ or exp micronesia/ or exp samoa/ or exp tonga/ or philippines/ or exp west indies/ |
| **Concept 3 : equation with keywords (17)** |  | ((("Low income" or "Middle income" or "Under-Developed" or "Third-World" or "Less-Developed" or Developing) adj2 (countr* or nation*)) or Afghanistan or Albania or Algeria or Angola or "Antigua and Barbuda" or Argentina or Armenia or Azerbaijan or Bangladesh or Belarus or Belize or Benin or Bhutan or Bolivia or Bosnia or Botswana or Brazil or "Burkina Faso" or Burundi or Cambodia or "Cabo Verde" or Cameroon or "Central African Republic" or Chad or China or Colombia or Comoros or Congo or "Costa Rica" or "Cote d’Ivoire" or Cuba or Korea or Congo or Dominica or "Dominican Republic" or Djibouti or Ecuador or "Equatorial Guinea" or Egypt or "El Salvador" or Eritrea or Eswatini or Ethiopia or Fiji or Gabon or Gambia or "Guinea-Bissau" or Georgia or Ghana or Grenada or Guatemala or Guyana or Haiti or Honduras or India or Indonesia or Iran or Iraq or Jamaica or Jordan or Kazakhstan or Kenya or Kiribati or Kosovo or Kyrgyzstan or Lao or Lebanon or Lesotho or Liberia or Libya or Iran or Madagascar or Malawi or Malaysia or Maldives or Mali or "Marshall Islands" or Mauritania or Mauritius or Mexico or Micronesia or Moldova or Mongolia or Montenegro or Montserrat or Morocco or Mozambique or Myanmar or Namibia or Nauru or Nepal or Nicaragua or Niger or Nigeria or Niue or "North Macedonia" or Pakistan or Palau or "Papua New Guinea" or Panama or "Paraguay" or Peru or Philippines or Rwanda or "Saint Helena" or "Saint Lucia" or "Saint Vincent and the Grenadines" or Samoa or "Sao Tome and Principe" or Senegal or "Sierra Leone" or "Solomon Islands" or Somalia or "South Africa" or "South Sudan" or "Sri Lanka" or "Suriname" or "Syrian Arab Republic" or Tajikistan or Tanzania or Thailand or "Timor-Leste" or Tokelau or Togo or Tonga or Tunisia or Turkey or Turkmenistan or Tuvalu or Uganda or Ukraine or Uzbekistan or Vanuatu or Venezuela or "Viet Nam" or "Wallis and Futuna" or "West Bank" or Yemen or Zambia or Zimbabwe).ab,kf,kw,ti. |
| **Concept 3 : mixed equation (18)** | **OR** | **(16 OR 17)** |
| **Concept 4 : equation with descriptors (19-20)** | 19  20 | Program Development/  models, educational/ |
| **21=19 OR 20** |  |  |
| **Concept 4 : equation with keywords (22)** |  | (Program* or Strateg* or Model* or Approach* or Project* or framework*).ab,kf,kw,ti. |
| **Concept 4 : mixed equation (23)** | **OR** | **(21 OR 22)** |
| **Final equation (S19)** | **AND** | **(12 AND 15 AND 18 AND 23)** |
| **LIMITATIONS** |  | **10 years (2011-current) and**  **languages (English and Spanish and French)** |
