# Supplementary material for: Continuing professional development (CPD) system development, implementation, evaluation and sustainability for healthcare professionals in low- and lower-middle-income countries: a rapid scoping review
Source: BMC Med Educ. 2023 Jul 6;23:498. doi: 10.1186/s12909-023-04427-6 (PMC10324177; doi:10.1186/s12909-023-04427-6)
Supplement: Supplementary file 3 — Additional file 3. Refinement of Inclusion and Exclusion Criteria. [file 12909_2023_4427_MOESM3_ESM.docx]

**Additional file 3: Refinement of Inclusion and Exclusion Criteria**

|  | Broad Criteria | Refined Criteria (exclusions added) |
| --- | --- | --- |
| Population | *Include*  **- Licensed healthcare workers:**  Audiologists, Chiropractors, Dental assistants, Dental hygienists, Dentists. Dietitians, Doctors, Genetic counsellors, , Licensed practical nurses, Medical laboratory technologists, Medical radiation technologists, Midwives, Nurses, Nursing assistants (licensed practical nurses, auxiliary nurse), Nutritionists, Occupational therapists, Opticians, Optometrists, Respiratory therapists, Paramedics, Pharmacists, Pharmacist technicians, Physicians, Physician assistants, Physiotherapists, Psychologists, Public health professionals, Speech-language pathologists, Social workers | *Inclusion same as broad criteria*  *New exclusions:*  - **Students in a preparatory program** (undergraduate, graduate and pre-service program)  - **Trainees in medical subspecialty**  - **Community care worker** or **skilled birthing persons** (not midwives)  - **Military health worker** |
| Location | *Include*  **- Under-developed, Low- and Middle-income countries:**  Afghanistan, Albania, Algeria, Angola, Antigua and Barbuda, Argentina, Armenia, Azerbaijan, Bangladesh, Belarus, Belize, Benin, Bhutan, Bolivia, Bosnia and Herzegovina, Botswana, Brazil, Burkina Faso, Burundi, Cambodia, Cabo Verde, Cameroon, Central African Republic, Chad, China, Colombia, Comoros, Congo, Costa Rica, Cote d’Ivoire, Cuba, Democratic People’s Republic of Korea, Democratic Republic of the Congo, Dominica, Dominican Republic, Djibouti, Ecuador, Equatorial Guinea, Egypt, El Salvador, Eritrea, Eswatini (Swaziland), Ethiopia, Fiji, Gabon, Gambia, Guinea-Bissau, Georgia, Ghana, Grenada, Guatemala, Guyana, Haiti, Honduras, India, Indonesia, Iran, Iraq, Jamaica, Jordan, Kazakhstan, Kenya, Kiribati, Kosovo, Kyrgyzstan, Lao People’s Democratic Republic, Lebanon, Lesotho, Liberia, Libya, Madagascar, Malawi, Malaysia, Maldives, Mali, “Marshall Islands”, Mauritania, Mauritius, Mexico, Micronesia, Moldova, Mongolia, Montenegro, Montserrat, Morocco, Mozambique, Myanmar, Namibia, Nauru, Nepal, Nicaragua, Niger, Nigeria, Niue, “North Macedonia”, Pakistan, Pala , “Papua New Guinea”, Panama, “Paraguay”, Peru, Philippines, Rwanda, “Saint Helena”, “Saint Lucia”, “Saint Vincent and the Grenadines”, Samoa, “Sao Tome and Principe”, Senegal, Serbia, “Sierra Leone”, “Solomon Islands”, Somalia, “South Africa”, “South Sudan”, "Sri Lanka", Suriname, “Syrian Arab Republic”, Tajikistan”, Tanzania, Thailand, “Timor-Leste”, Tokelau, Togo, Tonga, Tunisia, Turkey, Turkmenistan, Tuvalu, Uganda, Ukraine, Uzbekistan, Vanuatu, Venezuela, “Viet Nam”, “Wallis and Futuna”, “West Bank and Gaza Strip”, Yemen, Zambia, Zimbabwe | *Exclude from inclusion list:*  **- Upper-middle income countries:**  Albania, Algeria, Antigua and Barbuda, Argentina, Azerbaijan, Belarus, Belize, Bosnia and Herzegovina, Botswana, Brazil, China (People's Republic of), Colombia, Costa Rica, Cuba, Dominica, Dominica Republic, Ecuador, Equatorial Guinea, Fiji, Gabon, Grenada, Guyana, Iran, Iraq, Jamaica, Kazakhstan, Lebanon, Libya, Malaysia, Maldives, Marshall Islands, Mauritius, Mexico, Montenegro, Montserrat, Namibia, Nauru, Niue, North Macedonia, Palau, Panama, Paraguay, Peru, Saint Helena, Saint Lucia, Saint Vincent and the Grenadines, Samoa, Serbia, South Africa, Suriname, Thailand, Tonga, Turkey, Turkmenistan, Venezuela, Wallis & Futuna |
| CPD Program or System | *Include*  **- Descriptions of programs or systems defined as:**  Ongoing learning activities of healthcare professionals following graduation to maintain and develop a variety of knowledge and skills to meet the needs of patients and for the protection of the public (Combined definition from those provided by Fleet et al., 2008).  **- Descriptions of a CPD framework/model** | *Inclusions added:*  - **Descriptions of a broad CPD program** (national/system level)  - **Descriptions of a needs assessment for a broad CPD program**  *New exclusions:*  **- Descriptions of a CPD course (focused CPD) to address a specific clinical issue**  **- Descriptions of global health partnerships**  **- Descriptions of instrument development to measure CPD**  **- Assessments of knowledge, attitudes and practices related to a specific health issue.** |
| Publication year | *Include:*  ***-* Published within last 10 years (2012-2022)** | *Unchanged* |
| Publication language | *Include:*  **- Written in English, French or Spanish** | *Unchanged* |
