# Supplementary material for: Continuing professional development (CPD) system development, implementation, evaluation and sustainability for healthcare professionals in low- and lower-middle-income countries: a rapid scoping review
Source: BMC Med Educ. 2023 Jul 6;23:498. doi: 10.1186/s12909-023-04427-6 (PMC10324177; doi:10.1186/s12909-023-04427-6)
Supplement: Supplementary file 4 — Additional file 4. Characteristics of Included Articles and Grey literature. [file 12909_2023_4427_MOESM4_ESM.docx]

**Additional file 4: Characteristics of Included Articles and Grey literature**

**Characteristics of Included Articles**

| Reference^*^ | Objective | Country (LLMIC) | Health Professional | Appraisal |
| --- | --- | --- | --- | --- |
| Chekijian, S., et al. (2020) | To describe the phases of evolution of CME chronologically and details the legislative and regulatory framework surrounding each stage of development | Republic of Armenia a.k.a. Armenia | Physicians and non-physician health professionals | **Transparency:** Article is clearly written. **Completeness:** Provides sufficient information on the legal framework and evolution of CPD in Armenia but lacks information on the outcomes of the system or plans for evaluation. **Best practice:** Authors did not describe the process followed in the development of the report. Authors indicated that the input of stakeholders in various settings and at different time-points was sought to describe evolution of CPD in Armenia. |
| Chilomo, C., et al. (2014) | To describe the implementation and evaluation of changes made to the nursing CPD programme in Malawi supported by a 1-year ARC grant. | Malawi | Nurses and midwives | **Transparency:** The article is clear, structured, and easy to understand **Completeness:** More details could have been provided, especially on methodology, political process and policy. **Best practice:** The report development process was not described. |
| Clark, M., et al. (2015) | 1) To highlight nursing continuing education as a key initiative for strengthening healthcare delivery in low-resource settings, and 2) To provide an example of a nursing continuing education programme in Haiti | Haiti | Nurses | **Transparency:** Article is clearly written. **Completeness:** Provides sufficient information on the programme development, but lacks in description or comment on the regulatory, legal and accreditation framework put in place. **Best practice:** A process was briefly described to identify case examples of CPD in low-resources settings. Specific process for how the case study was conducted is lacking. |
| Dunleavy, K., et al. (2018) | To describe the use of a contextual instructional framework to guide the processes and instructional design choices for a series of continuing professional development courses for physiotherapists in Rwanda | Rwanda | Physiotherapists | **Transparency:** The article is clear and the level of detail provides confidence that the information is true. **Completeness:** Provides sufficient information to have an overall appreciation of the program developed and implemented; **Best practice:** The process used appears rigorous (e.g., Use of a framework; description of all steps; co-authors include Rwandan Physical Therapy association) |
| Iliffe, J. (2011) | To explore what some of the key features of a national CPD framework might be, drawing on the elements of other national programmes both inside and outside of Africa | Within Africa: Malawi, and South Africa | Nursing and midwifery | **Transparency:** The article is clear and the level of detail provides confidence that the information is true. **Completeness:** Gives good overview of elements, principles, definitions of different CPD systems. **Best practice**: The article is based on 'review' of CPD systems in different countries. |
| Li, Y., et al. (2017) | 1) To build, deploy, and evaluate an ICT4D (Information and Communication Technology for Development) solution called CMES (CME on a Stick) for the delivery and sharing of affordable and high- quality CME content for rural medical practitioners, focusing on Nepal as a test-bed, and 2) To continue the application and refinement of the Citizen-centric Capacity Development (CCD) framework for ICT4D | Nepal | Physicians | **Transparency:** Generally, the article is written clearly enough and was easy to understand. **Completeness:** Lacks some specificity regarding the individuals/groups referred to in the article. The article is an overview of the implementation process and lacked details. **Best practice:** Authors seemed to follow a framework that exhibited some level of "science" and rigor”. They engaged stakeholders (political and local) from the very beginning. |
| Li, Y., et al. (2020) | 1) To present a revised Citizen-centric Capacity Development (CCD) framework that focuses on goal driven ICT solution design and impact assessment, and 2) to investigate how the CCD framework guides the design, development, and assessment of CMES (CME on a Stick), a low-cost, integrative platform for the delivery of CME content to rural health workers in LICs. | Nepal | Physicians | **Transparency:** The article was easy to understand. **Completeness:** What is mostly missing is the evaluation of the CPD implementation (outcomes). Strengths include many details about the "technological" aspects of the CPD program, and the development of the CPD program were generally well described. The role of each stakeholder involved was less well described. **Best practice:** The authors followed and adapted a framework that exhibited a general scientific process, and they conducted qualitative research between each cycle, while engaging stakeholders from the very beginning (local and political). |
| Mack, H., et al. (2017) | To describe and discuss the advantages and disadvantages of five different approaches that can be used for implementing CPD programs in low-resource settings. | Non-specific | Physicians | **Transparency:** The article was easy to understand. **Completeness:** The article provides a thorough overview of the five different CPD program implementation approaches identified. **Best practice:** The article does not include a methods section so it is not clear how the five approaches or the supporting literature, were identified. However, the completeness of the information presented and the references cited suggest there was rigor to their process. |
| Michel-Schuldt, M., et al. (2018) | To describe a new model of CPD for midwives in Liberia. | Liberia | Nurses and midwives | **Transparency:**  The article is clearly written and provides sufficient detail about the current CPD model. **Completeness:**  More details about how the new model was developed and the implementation process for the pilot program would have contributed to the completeness. Similarly, indicators for the success of the pilot program could have been included. **Best practice:** Authors included stakeholders from Liberia. The process for developing the report was not described. |
| Moetsana-Poka, F., et al. (2014) | To describe the aims, development, implementation successes and challenges of a national CPD framework to improve the education and practice of nurses and midwives, and to strengthen the licensing regulatory capacity of the Lesotho nursing council. | Lesotho | Nurses and midwives | **Transparency:** The article is clear and the level of detail provides confidence that the information is true. **Completeness:** More details of framework would have strengthened the article. **Best practice**: Many authors were from Lesotho; The process was rigorous and well described. |
| Msibi, G. S., et al. (2014) | To describe the aim, development and implementation challenges and success of a national nursing and midwifery CPD framework in Swaziland. | Swaziland (Eswatini) | Nurses and midwives | **Transparency:** An interesting article that was well structured, clearly written, and easy to understand. **Completeness:** Sufficient information was presented describing the process for development of a national CPD framework. More information about the actual framework developed would have been beneficial. **Best practice:** A rigorous process was described by the authors. Authors were from Swaziland. |
| Shamim, S., et al. (2021) | To explore the existence and understanding of CPD in Ghana, Pakistan, and Trinidad and Tobago*, the barriers faced in development of a CPD model, and probable policies needed to build/improve such a model | Pakistan and Ghana  (**Trinidad and Tobago was not a LLMIC. Data about this country not extracted.)* | Pharmacists | **Transparency:** The article is clearly written. **Completeness:** The article provides sufficient information on the perceptions of pharmacists on CPD but limited in the number of participants who consented to participate; Unclear whether the views are broadly representative. **Best practice**: Study design and methods were described adequately. |
| Tyer-Viola, L. A., et al. (2013) | To discuss the need for continuing education for all practicing nurses and describes the implementation of a model for professional development of nurses in Bangladesh. | Bangladesh | Nurses | **Transparency:** The article is clearly written. **Completeness:** The article provides detailed information on the program being implemented (e.g., framework), but lacks description of challenges in implementation given the context in Bangladesh (e.g., barriers). Outcomes described are short-term and specific to the settings in this study. No discussion on whether a political process supported the partnership between the agency and healthcare institution. Also no description of accreditation and how it fits in to requirements for CPD for Bangladesh nurses. **Best practice:** A lack of description on the evaluation process (methods used for recruitment, data collection and analysis in evaluating the CPD framework.) |
| Undilashvili, A., et al. (2019) | To describe the evolution of CPD in Georgia. | Georgia | Physicians | **Transparency:** The article was difficult to follow, lacking in a clear purpose and headings. **Completeness**: There is missing information, it describes an overview of CPD in the country, including the stakeholders that were involved in the process however, few details are provided about the implementation and CPD system itself. **Best practice:** No details were provided about how the information was gathered. For example, there were needs, but how were they identified? The CPD was based on what framework? |
| Younes, N. A., et al. (2019) | To provide a better understanding of the state of CPD in the healthcare sector in Jordan with a view to informing the development of Jordan's first CPD framework. | Jordan | Physicians | **Transparency:** The article is written clearly and provides sufficient detail on the results of the study. **Completeness**: The article does not describe the implementation of the new CPD requirements/law (that came into effect in 2018) with sufficient depth. The article lacks some alignment with the discussion section, which focused on presenting different CPD requirements from other western countries. A description or proposal of a new framework for Jordan based on the results (as per the aim of the research conducted) would have strengthened this article. **Best practice:** The process for conducting the survey seemed rigorous although no data (psychometric data) about the data collection tool were presented. |

**Transparency:** *Is the article clear, structured, detailed enough and easy to understand?* **Completeness:** *Does the document provide sufficient information?* **Best practice:** *Was a rigorous process followed?*

*Abbreviations: ARC=African Health Regulatory Collaborative; CME=Continuing Medical Education; CPD: Continuing Professional Development; LIC=Low-income country; LLMIC=Low and lower-middle income country*

*^*^ References:*

Chekijian, S., Yedigaryan, K., Bazarchyan, A., Yaghjyan, G., & Sargsyan, S. (2021). Continuing medical education and continuing professional development in the republic of Armenia: the evolution of legislative and regulatory frameworks post transition. *Journal of European CME, 10*(1), 1853338. doi:<https://doi.org/10.1080/21614083.2020.1853338>

Chilomo, C., Mondiwa, M., & Wasili, R. (2014). Strengthening professional development in Malawi. *African Journal of Midwifery and Women's Health, 8*(1), 10-12. doi:<https://doi.org/10.12968/ajmw.2014.8.1.10>

Clark, M., Julmisse, M., Marcelin, N., Merry, L., Tuck, J., & Gagnon, A. (2015). Strengthening healthcare delivery in H aiti through nursing continuing education. *International Nursing Review, 62*(1), 54-63. doi: <https://doi.org/10.1111/inr.12165>

Dunleavy, K., Chevan, J., Sander, A. P., Gasherebuka, J. D., & Mann, M. J. (2018). Application of a contextual instructional framework in a continuing professional development training program for physiotherapists in Rwanda. *Disability and Rehabilitation, 40*(13), 1600-1608. doi: <https://doi.org/10.1080/09638288.2017.1300692>

Iliffe, J. (2011). Developing a national continuing professional development framework. *African Journal of Midwifery and Women's Health, 5*(4), 189-194. doi:<https://doi.org/10.12968/ajmw.2011.5.4.189>

Li, Y., Thomas, M. A., Rana, S. S., & Stoner, D. (2017). Continuing medical education on a stick: Nepal as a test bed. In J. Choudrie, Islam, M., Wahid, F., Bass, J., Priyatma, J. (Ed.), *Information and Communication Technologies for Development. ICT4D 2017.* IFIP Advances in Information and Communication Technology, vol 504. Springer, Cham. <https://doi.org/10.1007/978-3-319-59111-7_33>.

Li, Y., Thomas, M. A., Stoner, D., & Rana, S. S. (2020). Citizen-centric capacity development for ICT4D: the case of continuing medical education on a stick. *Information Technology for Development, 26*(3), 458-476. doi:<https://doi.org/10.1080/02681102.2020.1756730>

Mack, H. G., Golnik, K. C., Murray, N., & Filipe, H. P. (2017). Models for implementing continuing professional development programs in low-resource countries. *MedEdPublish, 6*(1). doi:<https://doi.org/10.15694/mep.2017.000018>

Michel-Schuldt, M., Dayon, M. B., Klar, R. T., Subah, M., King-Lincoln, E., Kpangbala-Flomo, C., & Broniatowski, R. (2018). Continuous professional development of Liberia's midwifery workforce—A coordinated multi-stakeholder approach. *Midwifery, 62*, 77-80. doi:<https://doi.org/10.1016/j.midw.2018.02.023>

Moetsana-Poka, F., Lehana, T., Lebaka, M., & McCarthy, C. F. J. (2014). Developing a continuing professional development programme to improve nursing practice in Lesotho. *African Journal of Midwifery and Women's Health, 8*(2), 10-13. doi:<https://doi.org/10.12968/ajmw.2014.8.Sup2.10>

Msibi, G. S., Mkhonta, N. R., Nkwanyana, N. R., Mamba, B., & Khumalo, G. T. (2014). Establishing a national programme for continuing professional development of nurses and midwives in Swaziland. *African Journal of Midwifery and Women's Health, 8*(2), 14-16. doi:<https://doi.org/10.12968/ajmw.2014.8.Sup2.14>

Shamim, S., & Rasheed, H. (2021). Continuing professional development for pharmacists in three countries with developing health systems. *Currents in Pharmacy Teaching and Learning, 13*(5), 471-478. doi:<https://doi.org/10.1016/j.cptl.2021.01.002>

Tyer-Viola, L. A., Timmreck, E., & Bhavani, G. (2013). Implementation of a continuing education model for nurses in Bangladesh. *The Journal of Continuing Education in Nursing, 44*(10), 470-476. doi:<https://doi.org/10.3928/00220124-20130816-07>

Undilashvili, A., Ebralidze, K., & Beriashvili, R. (2019). Continuous professional development of healthcare workers- analysis of the current state *Georgian Medical News*(297), 158-163. doi:PMID: 32011314

Younes, N. A., AbuAlRub, R., Alshraideh, H., Abu-Helalah, M. A., & Alhamss, S. (2019). Engagement of Jordanian physicians in continuous professional development: current practices, motivation, and barriers. *International Journal of General Medicine, 12*, 475. doi:10.2147/IJGM.S232248

**Characteristics of the Grey Literature**

| Source, (year) | Document title | Document type | Link | Objective | Country | Health Professional | Appraisal |
| --- | --- | --- | --- | --- | --- | --- | --- |
| The World Health Organization, (2015) | Regional Strategy on Strengthening Health Workforce Education and Training in South East Asia Region (2014-2019) | Guideline | <http://apps.who.int/iris/bitstream/handle/10665/160761/SEA-HSD-379.pdf?sequence=1> | To describe a regional strategy to strengthening health workforce education and training, including recommendations for CPD implementation | LLMICs in South-East Asia: Bangladesh, Bhutan, Democratic People's Republic of Korea, India, Indonesia, Myanmar & Nepal | Healthcare workers (including nurses) | **Currency:** Date specified in the document, and is within parameters set (last 10 years). **Relevancy:** Addresses education of HCW and specifically CPD systems' status in LLMICs in South-East Asia. **Accuracy**: References are provided. Process not described for how information was collected and collated to develop the report. **Authority:** Very credible (WHO) and appears on its official website. **Purpose:** Appears factual based on previous reports and each country’s "self-report" |
| Federal Ministry of Health Ethiopia, (2018) | Directive on Continuing Professional Development for Health Professionals | Ministry directive | <https://www.hu.edu.et/images/HuFiles/CPD/CPD_Guideline.pdf> | To describe the directives for CPD adopted by the Food, Medicine, and Healthcare Administration and Control Regulation for re-licensure and stipulates that health professionals shall be relicensed only when they have fulfilled the CEU required (as per the directive) | Ethiopia | All HCW involved in health services and licensed by the ministry or regional health regulatory bodies | **Currency:** Date specified in document and within set parameters (last 10 years). **Relevancy:** Reports on CPD directives for health workers in a LLMIC. **Accuracy:** Not known. Document is available on what appears to be a legitimate government website. **Authority:** Authored by a minister (or other representative). **Purpose:** Factual information is presented. |
| Fédération Internationale Pharmaceutique/ International Pharmaceutical Federation (2014) | Continuing Professional Development/ Continuing Education in Pharmacy: Global Report | Report | <https://www.fip.org/file/1407> | The document is a resource that resulted from discussions based on a literature review of the current information on CPD implementation and survey of countries (including quantitative and qualitative data) and case studies on the CPD, education and registration to assist countries in implementing CPD principles. Data was collected on 66 countries from African nations, Europe and Americas. | Not specific focus, but includes data from 66 African nations | Pharmacists | **Currency:** Date specified in the document and is within set parameters (within 10 yrs). **Relevancy:** Describes CPD status but not in any LLMIC specifically. Useful in providing information CPD framework described in an included published study in this review. **Accuracy:** References are provided and the data collection process is described. **Authority:** Legitimate authority (International pharmaceutical federation), copyrighted document, downloaded from the FIP website. **Purpose:** Provides factual information on global status of CPD for the pharmaceutical profession. |
| National Assembly of the Republic of Armenia, (2013) | Parliamentary hearings | News brief | <http://www.parliament.am/news.php?cat_id=2&NewsID=6352&year=%7B$Year%7D&month=%7B$Month%7D&day=%7B$Day%7D&lang=eng> | To document the partnership between the National Assembly of the Republic of Armenia standing committee on Healthcare, Maternity and Childhood with the USAID (funding support) for the investment into CPD system in Armenia. | Republic of Armenia | Physicians | **Currency:** Date specified in document and is within specified parameters (last 10 years). **Relevancy:** Reports on political process of CPD system for physicians and implementation in LLMIC. **Accuracy:** No specific references are made. **Authority:** Available on a legitimate government website. **Purpose:** Provides factual information. |
| European Union of Medical Specialists (EUMS), (n.d.) | The European Accreditation Council for CME (EACCME) | Webpage | <https://www.uems.eu/areas-of-expertise/cme-cpd/eaccme> | To co-ordinate a system to facilitate CME with national organizations where they exist. The EACCME is primarily responsible for structuring and accrediting CME-CPD activities to medical specialists throughout Europe | European union and other countries | Physicians | **Currency:** Unknown as no date is provided. However, documents on webpage are dated within last 5 years (2017). **Relevancy:** Provides information on CPD system and support in some LLMIC who have adopted this framework. **Accuracy:** Timeline is provided and information is reputable. Information is corroborated in other documents (published articles in this review). **Authority:** EACCME is well-established as is the umbrella organization (EUMC). **Purpose**: Provides factual information and resources. |
| Nurses and Midwives council of Malawi (NMCM), (n.d.) | Continuing CPD Brochure | Brochure | <http://www.nmcm.org.mw/wp-content/uploads/2023/03/CPD-Brochure.pdf> | Inform on CPD structure and requirements for all registered nurses and midwives in Malawi. | Malawi | Nurses and Midwives (including community midwifery assistants-CMAs) | **Currency:** No date on the document but website has a time stamp (2022).  **Relevancy:** Describes CPD requirements targeted to frontline health workers in LLMIC. **Accuracy:** Information is consistent with other documents on the website. **Authority:** No author presented. Identified on NMCM website, which appears legitimate. **Purpose:** Provide factual information about CPD requirement and process in that country. |
| Nurses and Midwives Council of Malawi (NMCM), (n.d.) | CPDC roles and responsibilities | Presentation | <http://www.nmcm.org.mw/downloads/> | To describe the attributes, roles and responsibilities of the CPD coordinator (CPDC), and the CPD committee role, structure and members. | Malawi | Nurses, Midwives, CMAs | **Currency:** No date on the document but website has a time stamp (2022). **Relevancy:** Describes role of CPD council for nurses and midwives in LLMIC. **Accuracy:** Information is consistent with other documents on the website. **Authority:** No author presented. Identified on NMCM, which appears legitimate.  **Purpose:** Provides factual information about CPD requirement and process in that country. |
| Nurses and Midwives council of Malawi (NMCM), (2009)  ^*^ Website date is 2022 | CPD coordinator job description | Document | <http://www.nmcm.org.mw/downloads/> | To describe qualifications, qualities desired, roles and responsibilities for the institution-level CPD coordinator (CPDC) | Malawi | Nurses, Midwives CMAs | **Currency:** Date listed as 2009, but website where it was found has timestamp of 2022, suggesting this document remains current. **Relevancy:** Describes job description for CPD coordinator in LLMIC. **Accuracy:** Information is consistent with other documents on the website. **Authority:** No author presented. Identified on NMCM. **Purpose:** Provide factual information about CPD requirement and process in that country. |
| Nurses and Midwives council of Malawi (NMCM), (n.d.) | CPD Checklist | Document | <http://www.nmcm.org.mw/downloads/> | To describe a checklist (process) for CPD implementation. To outline activities needed for CPD implementation and sustainability, identification of person responsible for implementation activities, tools needed, time frame and check box for completion of activity. | Malawi | Nurses, Midwives, CMAs | **Currency:** No date on the document but website has a time stamp (2022).  **Relevancy:** Describes CPD guide for CPD coordinator to implement CPD at the local level in a LLMIC **Accuracy:** Information is consistent with other documents on the website. **Authority:** No author presented. Identified on NMCM. **Purpose:** Provides factual information about CPD requirement and process in that country. |
| Nurses and midwives council of Malawi (NMCM), (2022) | Implementation of Continuing Professional Development (CPD) for nurses and midwives in Malawi | Presentation | <http://www.nmcm.org.mw/downloads/>  (CPD Policy for CPDC Training) | To provide an overview of the current CPD program for nurses and midwives in Malawi, highlighting changes made following the review of CPD policy and guidelines | Malawi | Nurses, Midwives, CMAs | **Currency:** Date suggests it is updated and current. **Relevancy:** Describes CPD requirements for CPD at the local level in LMIC. **Accuracy:** Information is consistent with other documents on the website. **Authority:** No author presented. Identified on nurses and midwives council of Malawi, which appears legitimate. **Purpose:** Appears to provide factual information about CPD requirement and process in that country. |
| Nurses and Midwives council of Malawi (MMCM), (n.d.) | CPDC Getting started | Document | <http://www.nmcm.org.mw/downloads/> | To provide a step by step approach to implement CPD in the workplace | Malawi | Nurses, Midwives, CMAs | **Currency:** No date on the document but timestamp on website (2022). **Relevancy:** Describes CPD council roles at the institutional level in LLMIC. **Accuracy:** Information is consistent with other documents on the website. **Authority:** No author presented. Identified on NMCM. **Purpose:** Provides factual information about CPD requirement and process in that country. |
| Nurses and midwives council of Malawi (NMCM), (n.d.) | Evidence Letter of CPD requirements fulfilled | Document | <http://www.nmcm.org.mw/downloads/> | To record CPD points accumulated by nurses/midwives at the institution. | Malawi | Nurses, Midwives, CMAs | **Currency:** No date on the document but timestamp on website (2022). **Relevancy:** Describes template to verify CPD requirements at the local level in LLMIC. **Accuracy:** Information is consistent with other documents on the website. **Authority:** No author presented. Identified on NMCM. **Purpose:** Provides factual information about CPD requirement and process in that country. |
| Rwanda Allied Health Professions Council, (n.d.) | CPD | Webpage and related tabs | <https://www.rahpc.org.rw/cpd> | To provide information for allied health workers on CPD system and requirements. | Rwanda | Physiotherapists and other allied health | **Currency:**  No specific date provided, but website is copyrighted 2018. **Relevancy:** Relevant as it describes the CPD requirements and process in Rwanda for Allied health professionals. **Accuracy:** No references are provided but there appears to be a significant amount of details on the requirements of CPD. **Authority:** Available on the council's website with links to other health professional websites in Rwanda. Contains contact information, telephone numbers and email addresses. However, some links appear to be broken. **Purpose:** Provides factual information. |
| Nepal Medical Council, (n.d.) | NPC Continuing professional development | Database | <https://cpdnmc.org.np/Account> | Data management system as a repository for evidenced-based education | Nepal | Physicians | **Currency:** Website is timestamped (2022) with updated events listed on the website. **Relevancy:** Provides information and portal to access CPD for Physicians (restricted to public) in Nepal. **Accuracy:** Little information is presented. **Authority**: Nepal Medical council. **Purpose:** Provides factual information |
| Liberian Board for Nurses and Midwifery, (n.d.) | Home, CPD/WCEA Project | Webpage | <https://lbnm.gov.lr/web/> | To provide registered nurses/midwives with the extensive educational digital library for CPD modules, information on COVID-19 and the data management program free of charge for Liberia. | Liberia | Nurses and Midwives | **Currency:** Website is not dated but date of latest news on website at the time of data extraction suggests it is current (March 2022). **Relevancy:** Provides information on CPD system and partnership with WCEA Liberia. **Accuracy:** Reference to legislation number is made, and information is consistent with that found on WCEA website. **Authority:** Liberian board for nurses and midwifery is a legitimate organization **Purpose:** Provides factual information for the audience. |
| One World Continuing Education Alliance (WCEA), (n.d.) | Homepage, projects and partners, ministries, professional councils associations and hospitals | Webpage | <https://oneworld.wcea.education/> | To significantly improve access to evidence-based education, developing skills and patient care, resulting in better health outcomes. | Afghanistan, Bangladesh*, Eswatini*, Ethiopia, Gambia, Georgia*, Ghana*, Haiti*, India, Indonesia, Jordan*, Kenya, Lesotho*, Liberia*, Malawi*, Myanmar, *Nepal*, Nigeria, Pakistan, Rwanda***,** Sierra Leone, Sudan, Sri Lanka, Tanzania, Turkey, Uganda, Zambia, Zimbabwe (**countries identified in included published literature for this review)* | Nurses, midwives, physicians | **Currency:** News presented on website is updated (April 2022). **Relevancy:** Provides information on partnership with LLMIC to provide CPD support (activities and LMS platforms). **Accuracy:** Information corresponds to that found on other websites (LLMIC nursing association websites). **Authority:** Appears to be legitimate as an organization, listed on other websites. **Purpose:** Provides information to market its services. |
| African health professional regulatory collaborative for nurses and midwives, (n.d.) | A Continuing Professional Development Framework for Lesotho | Presentation | <http://www.commonwealthnurses.org/Documents/LesothoPresentationDraftCPDframework.pdf> | Outlines the CPD framework for Lesotho. | Lesotho | Nurses and Midwives | **Currency:** Date not indicated. Unclear when this document was produced. **Relevancy:** Provides a description of the CPD system and framework in Lesotho. **Accuracy:** Information consistent with peer-reviewed literature. **Authority:** Includes reputable acknowledgements on title slide including ARC, CDC, Emory University etc. **Purpose:** Provides factual information on CPD in Lesotho. |
| Ministry of Health Lesotho, (n.d.) | Nursing Directorate | Report | <http://health.gov.ls/nursing-directorate/> | To describe the functions of the department of the nursing directorate as part of the Ministry of Health of Lesotho | Lesotho | Nurses and Midwives | **Currency:** No date on document. Downloaded from website with timestamp 2021. **Relevancy:** Describes some information on Lesotho government role on CPD. **Accuracy:** No other references provided, but information is presented coherently. **Authority:** Downloaded from official government website. **Purpose:** Provides factual information. |
| Nursing Eswatini Council, (n.d.) | Frameworks 6. Continuing professional development | Webpage | <https://www.eswatininursingcouncil.com/#/frameworks> | To describe CPD requirements for nurse and midwife members. | Eswatini (Swaziland) | Nurses and midwives | **Currency:** Website copyright date is 2021. **Relevancy:** Provides an overview of CPD in Eswatini. **Accuracy:** Appears to be accurate though no reference provided. Information clearly presented. **Authority:** Downloaded from Eswatini nursing council website, which appears legitimate. **Purpose:** Provides factual information. |
| Nursing and Midwifery Council Ghana, (2016) | Continuing professional development PROGRAMMES FOR NURSE ASSISTANTS, NURSES AND MIDWIVES | Report | <https://ces-assets.s3.amazonaws.com/public/documents/CPD-Guideline-Nurse-Assistants-Nurses-Midwives.pdf> | Outlines the CPD system in Ghana. | Ghana | Nurses, midwives, nursing assistants | **Currency:** Dated within set parameters set for this review (10 years). **Relevancy:** Provides overview of CPD system in LLMIC. **Accuracy:** No specific reference mentioned but information is complete and presented clearly. **Authority:** Nursing and midwifery council of Ghana. **Purpose:** Provides factual information |
| Pakistan Nursing Council, (n.d.) | Launching of continuing professional development program | Webpage | <https://www.pnc.org.pk/Free_CPD_Courses.htm> | To provides free resources using the WCEA platform (web and mobile app) with the LMS to maintain a log and download certificates. | Pakistan | Nurses and midwives | **Currency:** No date provided. **Relevancy:** Provides minimal information on the launch of CPD system and partnership to delivery CPD activities in LLMIC. **Accuracy:** Linked with ICN, WCEA and seems to present information that is complete. Website was developed with support from USAID. **Authority:** Webpage is on the Pakistan Nursing council website. **Purpose**: Provides factual and instructive information to members. |
| AK Khan Healthcare Trust, (n.d.) | Nursing Bridge Program | Webpage | <http://akkhanhealthcaretrust.org/our-programmers/nursing-bridge-programme-nbp/> | To provide a year long program for practicing nurses in Bangladesh, to develop new skills and aims to enable existing nurses in Bangladesh to attain competitive standards through improved clinical knowledge, explores to latest innovations and development of critical thinking and evidence-based practice. | Bangladesh | Nurses | **Currency:** No date specifically on post. Website has timestamp (2017), which is within set parameters. **Relevancy:** Provides some information on CPD activities in Bangladesh for nurses. **Accuracy:** No references are provided. Presents information clearly, no reason to suggest information is inaccurate. **Authority:** AK Khan is well known with collaborations with US based non-profit organizations and hospitals. **Purpose:** Provides factual information on program. |
| USAID Jordan, (2018) | Factors influencing CPD effectiveness and practices | Report | <https://jordankmportal.com/resources/factors-influencing-cpd-effectiveness-and-practices-in-the-healthcare-sector-in-jordan> | To describe a mixed methods study to review factors that influence CPD offerings, needs, practices, experiences and effectiveness in the healthcare sector in Jordan, across different professional groups. | Jordan | Physicians, dentists, pharmacists, nurses, and allied healthcare professionals | **Currency:** Dated within the set parameters. **Relevancy:** Relevant as it describes needs assessment for CPD in a LLMIC. **Accuracy:** References are provided as well as detailed process for data collection and presentation of findings. **Authority:** Authors are described and produced in conjunction with USAID. **Purpose:** Provides factual information on CPD system in Jordan |

**Currency:** *How current is the information? Is the information current enough for the topic? When was the page created, or last updated? Is there a copyright date on the page?* **Relevancy:** *Is the information relevant to the topic? Who is the target audience for this website?* **Authority:** *Who created the site or the page? Is it an individual, a corporation, an organization or association? What is known about the creator of the website? Are they credible and reputable? Are there ads on the site? If so, do the ads relate at all to the content of the website?* **Accuracy:** *Are sources or references given to support the information or evidence provided? Are there spelling or grammar errors? What information is provided about the author and his or her credentials, experience or education? Can you find similar or the same information in other credible sources?* **Purpose:** *Is the information provided factual only, or is it opinion or biased? Is the website trying to sell something, or persuade toward a particular point of view?* (Adapted from CRAAP test, Sheridan College Library Guides <https://sheridancollege.libguides.com/c.php?g=701210&p=5054766>)

*Abbreviations: ARC=African Health Regulatory Collaborative; CDC= Centers for Disease Control and Prevention; CEU=Continuing Education Units; CMA= Community Midwife Assistant; CME=Continuing Medical Education; CPD: Continuing Professional Development; CPDC=Continuing Professional Development Coordinator; EACCME= The European Accreditation Council for CME; EUMS=European Union of Medical Specialists; HCW= Healthcare worker; ICN= International Council of Nurses; LLMIC=Low and lower-middle income country; LMS=Learning Management System; WCEA=One World Continuing Education Alliance;*
