# Supplementary material for: Continuing professional development (CPD) system development, implementation, evaluation and sustainability for healthcare professionals in low- and lower-middle-income countries: a rapid scoping review
Source: BMC Med Educ. 2023 Jul 6;23:498. doi: 10.1186/s12909-023-04427-6 (PMC10324177; doi:10.1186/s12909-023-04427-6)
Supplement: Supplementary file 5 — Additional file 5. Bangladesh TWG-CPD action plan and timeline, July 2022. [file 12909_2023_4427_MOESM5_ESM.docx]

**Additional file 5: Bangladesh TWG-CPD action plan and timeline, July 2022**

| **#** | **ACTION** | **STEPS** | **TIMELINE** |
| --- | --- | --- | --- |
| **1** | Establish leadership and mobilize key stakeholders for the process | 1. Identify the leadership for CPD and especially linking with license renewal (BNMC, DGNM)    1. BNMC are concerned with the implementation process however they are key       1. Meet with BNMC (DGNM will meet with the registrar in the BNMC to discuss the project       2. BNMC is central to establishing the CPD system in Bangladesh but staff shortages are cause for concern 2. Other stakeholders wanted to have CPD linked to relicensing and could be involved in supporting BNMC    1. DGNM is motivated and available (main stakeholder)       1. Could be available for the provision of CPD (BNA could be available)    2. Concern with the accessibility of CPD activities (Online CPD?)    3. CPD linked with the career path? | **Priority / Before September 2022** |
| **2** | Develop a framework and outline of CPD requirements and guideline | 1. Determine all elements that will be detailed in the guideline    1. Point attribution per type of CPD    2. Required categories of CPD    3. Total number of points to compile in 5 years    4. Distribution of CPD during the 5 years (how many points/year? How to distribute the CPD throughout the 5 years)    5. Documentation process of CPD activities       1. Online option? To be developed       2. Paper (CPD portfolio or logbook)    6. Evaluation process    7. Monitoring system    8. Penalties if non-compliance? 2. Bangladesh recently adopted a *National Lifelong learning Policy*. Definition of “lifelong learning” in line with CPD and should be consulted to make sure that Policy and guidelines support each other. | **Fall 2022** |
| **3** | Draft a CPD guideline included in relicensing policy | Work with TWG-CPD to draft a guideline and relicensing policy based on reference documents and decisions from Action #2 | **Fall 2022** |
| **4** | Plan the financing with main stakeholders (MoHFW, DGNM and BNMC) | … According to action # 2 decisions and current action plan, create a budget for CPD system launch and implementation. | **Fall 2022** |
| **5** | Create a CPD unit (DGNM/BNMC/BNA) | Unit will plan CPD offer and delivery, manage CPD related activities, and monitoring of CPD guideline implementation   1. Draft ToR/mandate for the CPD unit 2. Identify members/staff of the CPD unit 3. Establish funding for the CPD unit | **Immediately after getting approval** |
| **6** | Conduct a needs assessment to identify attitudes, motivation, preferences, barriers to CPD participation (healthcare professionals and stakeholders) | 1. Gathering Statistical information on the nursing workforce is necessary to determine the CPD requirements to include in the guidelines    1. Who are the nurses? Their employers, their specialties, number of years of experience, Basic nursing training (Diploma, BSc, etc.) (some information available in the DGNM database)       1. Ask questions during the relicensing process about nurse satisfaction with the current relicensing process       2. Some stats:          1. 44,000 government deployed (CPD activities for them)          2. 12,000 BSc/MSc/MPH/PhD out of 44,000    2. Who will benefit from what CPD - (Diploma vs BSc vs MSc or other)    3. Identify priority areas for priority groups of nurses 2. Survey of nurses in different work context and sectors (Online survey monkey?) | **Fall 2022** |
| **7** | Inventory existing CPD offer/opportunities and plan for the creation and implementation of complementary training based on needs assessment | 1. Plan for the creation of an online CPD platform under the leadership of DGNM, BNMC & BNA, social media 2. Identify national or international sources of CPD - using the resources like video, manual, clinical practice guideline, different apps. Online and onsite training offering from DGHS, DGFP, DGNM & other government & non-Government organization.    1. Assess availability and recognition of a diversity of CPD activities for credits/hours | **Fall 2022** |
| **8** | Define outcomes / categories of CPD based on needs assessment survey (step 5b) and the development /evolution of health care (updating /developing new skills to keep-up) and determine the process for evaluation | 1. Define expected CPD system outcomes based on:    1. CPD definition    2. Needs assessment (statistical data and survey)    3. Health care priorities of Bangladesh 2. Identify CPD categories and progression according to:    1. Career paths    2. Health care priorities of Bangladesh    3. Needs assessment (statistical data and survey) 3. Evaluation:    1. Define an evaluation strategy based on expected outcomes    2. Plan a budget for CPD system evaluation | **January – March 2023** |
| **9** | Identify and/or develop CPD activities and materials (online and in-person) | 1. Identify areas of need for CPD module development based on identified CPD categories and progression (action # 8) according to:    1. Career paths    2. Health care priorities of Bangladesh    3. Needs assessment (statistical data and survey) 2. Plan the development of modules according to needs    1. Identify who/when/how the modules will be developed    2. Plan the financing of the module development | **January – March 2023** |
| **10** | Construct an accreditation structure | 1. Identify main elements of accreditation structure    1. Criteria to be examined by accreditation organisation 2. Identify one or more authorized accreditation organisations    1. Existing international accrediting organisations    2. Local organisation(s)    3. Other? 3. Determine accreditation process for:    1. In person CPD    2. Online CPD    3. Other? | **January - March 2023** |
| **11** | Develop a implementation and monitoring system for CPD system and link to license renewal | 1. Identify/develop tools for documenting and adding CPD requirements to the current license renewal procedure    1. DGNM & BNMC already have different online accessibility like digital registration system, registration renewal, admission & incorporate online courses for CPD) easily accessible, low cost and convenient times       1. Determine which system will be the basis       2. Create/modify existing system to include CPD requirements       3. Identify and train the person/team who will operate this system    2. Decide if/how online CPD is also accessible through the same organisation/system with partial funding through increase of relicensing fees       1. If not, who will be the provider and make sure Online and other CPD providers can be found by nurses who are looking for CPD 2. Identify/ develop tools for the implementation of the CPD system monitoring strategy developed in the guideline/policy document    1. Data collection and statistical analysis       1. What indicators       2. What frequency       3. Etc.    2. Monitoring of finances | **January – March 2023** |
| **12** | Mount and launch a communication strategy to promote CPD (for nurses, employers, providers, etc.) uptake and compliance | Motivation and sensitization through:   1. stakeholder meeting division wise; 2. training on CPD; 3. arranging national network meeting in education & service sector; 4. social media strategy; 5. develop CPD poster, brochure & so on. | **Before and after the launch of the CPD system implementation** |
| **13** | Evaluate the CPD system | 1. Process evaluation, example: man, money, materials 2. Product/outcome evaluation 3. Determine and implement a strategy for improving CPD system according to evaluation findings | **After beginning of implementation** |

*BNA= Bangladesh nurses association; BNMC= Bangladesh Nursing and Midwifery Council; CPD= Continuing Professional Development; DGNM= Directorate General of Nursing and Midwifery; MoHFW= Ministry of Health and Family Welfare; ToR= Terms of reference; TWG= Technical Working Group*
